# Near-Infrared Aggregation-Induced Emission-Active Probe Enables *in situ* and Long-Term Tracking of Endogenous β-Galactosidase Activity

**DOI:** 10.3389/fchem.2019.00291

**Published:** 2019-05-14

**Authors:** Wei Fu, Chenxu Yan, Yutao Zhang, Yiyu Ma, Zhiqian Guo, Wei-Hong Zhu

**Affiliations:** State Key Laboratory of Bioreactor Engineering, Shanghai Key Laboratory of Functional Materials Chemistry, School of Chemistry and Molecular Engineering, Institute of Fine Chemicals, East China University of Science and Technology, Shanghai, China

**Keywords:** fluorescent probe, near-infrared, aggregation-induced emission, β-galactosidase, *in situ*, long-term tracking

## Abstract

High-fidelity tracking of specific enzyme activities is critical for the early diagnosis of diseases such as cancers. However, most of the available fluorescent probes are difficult to obtain *in situ* information because of tending to facile diffusion or inevitably suffering from aggregation-caused quenching (ACQ) effect. In this work, we developed an elaborated near-infrared (NIR) aggregation-induced emission (AIE)-active fluorescent probe, which is composed of a hydrophobic 2-(2-hydroxyphenyl) benzothiazole (HBT) moiety for extending into the NIR wavelength, and a hydrophilic β-galactosidase (β-gal) triggered unit for improving miscibility and guaranteeing its non-emission in aqueous media. This probe is virtually activated by β-gal, and then specific enzymatic turnover would liberate hydrophobic AIE luminogen (AIEgen) QM-HBT-OH. Simultaneously, brightness NIR fluorescent nanoaggregates are *in situ* generated as a result of the AIE-active process, making on-site the detection of endogenous β-gal activity in living cells. By virtue of the NIR AIE-active performance of enzyme-catalyzed nanoaggregates, QM-HBT-βgal is capable of affording a localizable fluorescence signal and long-term tracking of endogenous β-gal activity. All results demonstrate that the probe QM-HBT-βgal has potential to be a powerful molecular tool to evaluate the biological activity of β-gal, attaining high-fidelity information in preclinical applications.

## Introduction

Specific enzymes play vital roles in a wide range of biological processes. Among them, β-galactosidase (β-gal) is overexpressed in primary ovarian cancers, which has been regarded as an important biomarker for cell senescence and ovarian cancer diagnosis (Dimri et al., 1995; Spergel et al., 2001). In view of this importance, real-time tracking of β-gal activity has become a powerful tool for accurate disease diagnostics. Recently, fluorescent probes have gained ever-increasing attention owing to its noninvasiveness to tissue and high sensitivity (Sun et al., 2016; Xu K. et al., 2016). However, current strategy for enzyme probes is generally based on fluorophores that are soluble in the cytoplasm (Bhuniya et al., 2014; Li X. et al., 2014; Zhang et al., 2014, 2018; Wang F. et al., 2015; Xu et al., 2015; Makukhin et al., 2016; Wu et al., 2016; Chen X. et al., 2017). These responsive probes largely suffer from inaccurate *in situ* information about biocatalytic activity, because the products of small molecules by enzyme conversion quickly diffuse away from the site of their generation (Kamiya et al., 2011; Yang et al., 2013; Li L. et al., 2014; Yin et al., 2014; Xu Q. et al., 2016; Zhou et al., 2016; Zhu et al., 2016; Wu et al., 2017). These released fluorophores even tend to translocate out of cells, thus making long-term tracking in living subjects difficult (Taylor et al., 2012; Wang et al., 2013; Liu H. W. et al., 2017). On the other hand, it is still far from achieving *in situ* accurate information, owing to the distorted signal from inevitable aggregation-caused quenching (ACQ) effect (Sun et al., 2014; Wu et al., 2014; Li et al., 2015; Gu et al., 2016; Liu Z. et al., 2017; Qi et al., 2018). Therefore, it is an urgent demand to overcome the dilemma of the released fluorophores between aggregation requirement for diffusion-resistant and ACQ effect resulting from aggregation.

With this in mind, we envisioned that near-infrared (NIR) aggregation-induced emission (AIE) probes (Qin et al., 2012; Leung et al., 2013; Mei et al., 2015; Guo et al., 2016; Wang et al., 2016; Yan et al., 2016; Liu L. et al., 2017; Shi et al., 2017; Yang et al., 2017; Zhang F. et al., 2017; Feng and Liu, 2018; Wang Y.-L. et al., 2018; Xie et al., 2018) can provide reliable opportunities to address the aforementioned intractable dilemma. The design of the AIE fluorophores extending into NIR wavelength for decreased autofluorescence and high penetration depth is essentially required for attaching additionally a hydrophobic π-conjugated bridge (Guo et al., 2014; Lim et al., 2014; Chevalier et al., 2016; Andreasson and Pischel, 2018; Li et al., 2018; Yan et al., 2018a,b,c). Impressively, nanoaggregates of the released fluorophores ideally meet the hydrophobic requirements for long-term tracking, and the AIE character of the aggregates can well solve the notorious ACQ effect. Furthermore, we anticipate that AIE-active β-gal probes integrating light-up NIR characteristic in synergy with tunable aggregation behavior could make a breakthrough to detect endogenous β-gal with high-fidelity imaging in living subjects. During the response to β-gal, the aggregation behavior of the AIE probe altered from the molecular dissolved state into the aggregated state, achieving AIE-active NIR mode. In this case, the more AIEgens aggregate, the brighter their NIR emission becomes, making them suitable for *in situ* sensing and long-term tracking of biomolecules in living systems (Kwok et al., 2015; Peng et al., 2015; Yuan et al., 2016; Nicol et al., 2017). However, as far as we know, AIE-active β-gal probes possessing the characteristics of both localizable NIR fluorescence signal and long-term tracking mode are scarcely reported.

**Scheme 1:**
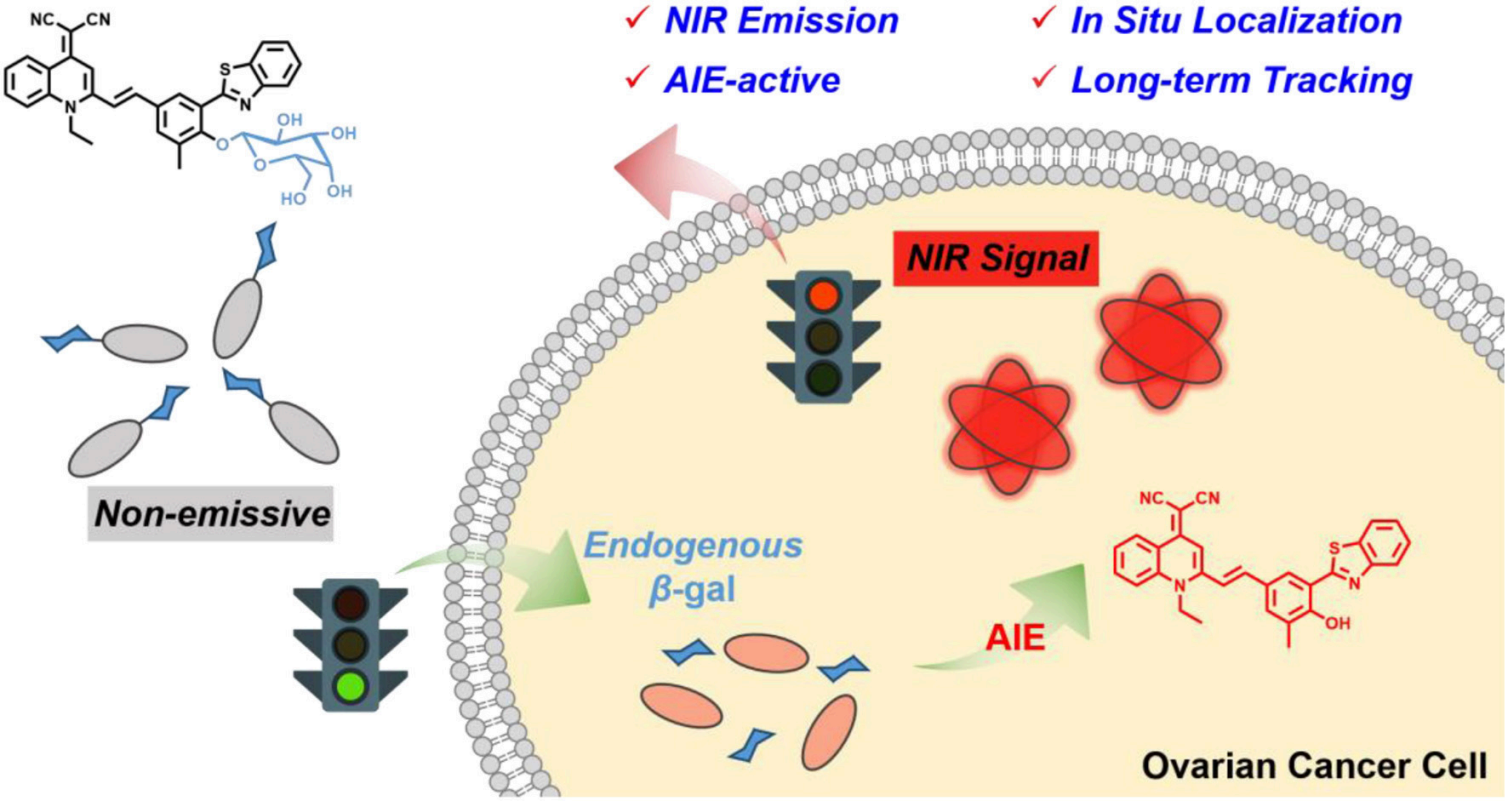
Schematic illustration of an enzyme β-gal-regulated liberation strategy for on-site sensing and long-term tracking.

Herein, we developed an elaborated NIR AIE-active β-gal probe for enabling *in situ* and long-term tracking of endogenous enzyme activity (Scheme 1). Firstly, we focus on our group-developed AIE building block of quinoline-malononitrile (QM) to overcome the enrichment quenching effect (Shi et al., 2013; Shao et al., 2014, 2015; Wang M. et al., 2018). Then, the lipophilic 2-(2-hydroxyphenyl) benzothiazole (HBT) moiety is covalently attached as an external π-conjugated backbone for extending the NIR emission. Furthermore, the masking of the phenolic hydroxyl group prohibits the excited-state intramolecular proton transfer (ESIPT) process and thus largely suppresses fluorescence (Kwon and Park, 2011; Thorn-Seshold et al., 2012; Hu et al., 2014; Zhou et al., 2015; Cui et al., 2016; Chen L. et al., 2017; Chen Y. H. et al., 2017; Zhang P. et al., 2017; Sedgwick et al., 2018a,b; Zhou and Han, 2018). Finally, we utilized the hydrophilic galactose moiety as the β-gal-triggered unit for keeping probes in the fluorescence-*off* state with minimal background. When converted by β-gal, the probe releases free QM-HBT-OH, which is found to be nearly insoluble and aggregated in water and shows bright NIR fluorescence owing to the AIE building block with extended π-conjugated structure. By virtue of the NIR AIE-active performance of enzyme-catalyzed nanoaggregates, QM-HBT-βgal is capable of affording a localizable fluorescence signal and long-term tracking of endogenous β-gal activity. Our results demonstrate that the probe QM-HBT-βgal has potential to be a powerful molecular tool to evaluate the biological activity of β-gal.

## Experimental Section

### Materials and General Methods

Unless especially stated, all solvents and chemicals were purchased from commercial suppliers in analytical grade and used without further purification. β-Galactosidase (β-gal) was supplied by *J&K* Scientific Ltd (Beijing, China). The ^1^H and ^13^C NMR spectra were recorded on a Bruker AM 400 spectrometer, using TMS as an internal standard. High-resolution mass spectrometry data were obtained with a Waters LCT Premier XE spectrometer. Absorption spectra were collected on a Varian Cary 500 spectrophotometer, and fluorescence spectra measurements were performed on a Varian Cary Eclipse fluorescence spectrophotometer. The time-dependent fluorescence measurements were conducted upon continuous illumination (Hamamatsu, LC8 Lightningcure, 300 W). Dynamic light scattering (DLS) experiments were conducted with Zetasizer Nano-ZS (Malvern Instruments, Worcestershire, UK), and scanning electron microscope (SEM) images were operated on a JEOL JSM-6360 scanning electron microscope. Confocal fluorescence images were taken on a confocal laser scanning microscope (CLSM, Leica confocal microscope TCS SPS CFSMP).

### General Procedure for *in vitro* Monitoring β-gal Activity

Probes were dissolved in dimethyl sulfoxide (DMSO, AR) to obtain 1-mM stock solutions. All UV–vis absorption and fluorescence spectra measurements were carried out in PBS/DMSO buffer solution (7:3, v/v, pH = 7.4, 50 mM). In a 3-mL tube, PBS buffer (2.1 mL) and DMSO (900 μL) solution were mixed, and then the probe (30 μL) was added to obtain a final concentration of 10 μM. β-Gal was dissolved in a PBS buffer, and an appropriate volume was added to the sample solution. After rapid mixing of the solution, it was transferred to a 10 × 10-mm quartz cuvette and incubated at 37°C for *in vitro* detection.

### Cell Experiment

#### Cell Lines

This study was performed in strict accordance with ethical standards including ethics committee approval and consent procedure, and adhered to standard biosecurity and institutional safety procedures. This study was performed in strict accordance with the NIH *Guidelines for the Care and Use of Laboratory Animals* (NIH Publication No. 85-23 Rev. 1985) and was approved by the Institutional Animal Care and Use Committee of National Tissue Engineering Center (Shanghai, China).

The human ovarian adenocarcinoma cell line SKOV-3 cells and the human epithelioid cervical carcinoma cell line HeLa cells were purchased from the Institute of Cell Biology (Shanghai, China). Cells were all propagated in T-75 flasks cultured at 37°C under a humidified 5% CO_2_ atmosphere in RPMI-1640 medium or DMEM medium (GIBCO/Invitrogen, Camarillo, CA, USA), which were supplemented with 10% fetal bovine serum (FBS, Biological Industry, Kibbutz Beit Haemek, Israel) and 1% penicillin–streptomycin (10,000 U mL^−1^ penicillin and 10 mg mL^−1^ streptomycin, Solarbio Life Science, Beijing, China).

#### *In vitro* Cytotoxicity Assay

The cell cytotoxicity of QM-HBT-βgal to SKOV-3 cells and HeLa cells was measured by 3-(4,5-dimethylthiazol-2-yl)-2,5-diphenyltetrazolium bromide (MTT) assay. Cytotoxicity was evaluated by Cell Counting Kit-8 (Dojindo, Tokyo, Japan) according to the factory's instruction. Cells were plated in 96-well plates in 0.1-mL volume of DMEM or RPMI-1640 medium with 10% FBS, at a density of 1 × 10^4^ cells/well, and added with desired concentrations of SKOV-3. After incubation for 24 h, absorbance was measured at 595 nm with a Tecan GENios Pro Multifunction Reader (Tecan Group Ltd., Maennedorf, Switzerland). Each concentration was measured in triplicate and used in three independent experiments. The relative cell viability was calculated by the following equation: cell viability (%) = (OD_treated_/OD_control_) × 100%.

#### Cells Imaging

Cells were seeded onto glass-bottom petri dishes in culture medium (1.5 mL) and allowed to adhere for 12 h before imaging. Probe QM-HBT-βgal at a final concentration of 10 μM (containing 0.1% DMSO) was added into culture medium and incubated for different times at 37°C under a humidified 5% CO_2_ atmosphere. Cells imaging was captured by using a confocal laser scanning microscope (Leica confocal microscope TCS SPS CFSMP) with a 60 × oil immersion objective lens. The fluorescence signals of cells incubated with probes were collected at 650–700 nm under excitation wavelength at 460 nm.

### Synthesis of Probe QM-HBT-βgal

#### Synthesis of Compound 1

A mixture of 2-aminobenzenethiol (2 g, 16.0 mmol) and 2-hydroxy-3-methylbenzoic acid (2 g, 16.0 mmol) in polyphosphoric acid (15 mL) was heated in an oil bath at 180°C for 10 h under an argon atmosphere and then was cooled at room temperature. The mixture was then poured into ice, filtered, and the resulting solid product was washed with water, dried in air, and finally purified by column chromatography using dichloromethane/petroleum ether (v/v, 2:1) as the eluent to afford a white solid product (1 g): yield 26%. ^1^H NMR (400 MHz, CDCl_3_, ppm): δ = 2.36 (s, 3H, C***H***_3_-H), 6.87 (t, *J* = 8.0 Hz, 1H, phenyl-H), 7.27 (s, 1H, phenyl-H), 7.41 (t, *J* = 8.0 Hz, 1H, phenyl-H), 7.50 (t, *J* = 8.0 Hz, 1H, phenyl-H), 7.56 (d, *J* = 8.0 Hz, 1H, phenyl-H), 7.90 (d, *J* = 8.0 Hz, 1H, phenyl-H), 7.97 (d, *J* = 8.0 Hz, 1H, phenyl-H), 12.8 (s, 1H, -O***H***). ^13^C NMR (100 MHz, CDCl_3_, ppm): δ 169.81, 156.29, 151.83, 133.69, 132.71, 126.63, 126.07, 125.43, 122.07, 121.49, 119.00, 116.02, 16.08. Mass spectrometry (ESI-MS, *m/z*): [M + H]^+^ calcd. for [C_14_H_11_NOS + H]^+^ 242.0640; found 242.0636.

#### Synthesis of Compound 2

Compound 1 (200 mg, 0.83 mmol) was dissolved in 10 mL of trifluoroacetic acid, and then, hexamethylenetetramine (174.5 mg, 1.25 mmol) was added. The mixture was heated in an oil bath at 90°C for 20 h under an argon atmosphere. The reaction mixture was then cooled to room temperature and poured into 6 M HCl (30 mL) and extracted with CH_2_Cl_2_. The combined organic extracts were washed with saturated brine. Next, purification was done by column chromatography using dichloromethane as the eluent to afford the pure product as a whiteness solid (100 mg): yield 45%.^1^H NMR (400 MHz, CDCl_3_, ppm): δ 2.42 (s, 3H, C***H***_3_-H), 7.47 (t, *J* = 8.0 Hz, 1H, phenyl-H), 7.55 (t, *J* = 8.0 Hz, 1H, phenyl-H), 7.80 (s, 1H, phenyl-H), 7.95 (d, *J* = 8.0 Hz, 1H, phenyl-H), 8.01 (d, *J* = 8.0 Hz, 1H, phenyl-H), 8.11 (d, *J* = 8.0 Hz, 1H, phenyl-H), 9.92 (s, 1H, C***H***O-H), 13.6 (s, 1H, -O***H***). ^13^C NMR (100 MHz, CDCl_3_, ppm): δ 190.29, 168.63, 161.12, 151.27, 133.98, 132.62, 128.95, 128.18, 126.98, 126.02, 122.20, 121.67, 116.17, 16.06. Mass spectrometry (ESI-MS, *m/z*): [M – H]^−^ calcd. for [C_15_H_11_NO_2_S – H]^−^ 268.0432; found 268.0436.

### Synthesis of QM-HBT-OH

Compound 2 (100 mg, 0.37 mmol) and 2-(1-ethyl-2-methylquinolin-4(1*H*)-ylidene)malononitrile (132 mg, 0.56 mmol) were dissolved in acetonitrile (20 ml) with piperidine (0.5 ml). Then, the mixture was refluxed for 10 h under an argon atmosphere. The solvent was removed under reduced pressure, and then, the crude product was purified by silica gel chromatography using dichloromethane as the eluent to afford QM-HBT-OH as a red solid (50 mg): yield 28%. ^1^H NMR (400 MHz, DMSO-*d*_6_, ppm) δ1.45 (t, *J* = 8.0 Hz, 3H, NCH_2_*C**H***_3_-H), 2.18 (s, 3H, C***H***_3_-H), 4.59 (q, *J* = 8.0 Hz, 2H, N*C**H***_2_CH_3_-H), 7.09 (d, *J* = 16.0 Hz, 1H, alkene-H), 7.15 (s, 1H, phenyl-H), 7.29 (t, *J* = 8.0 Hz, 1H, phenyl-H), 7.42 (d, *J* = 8.0 Hz, 1H, phenyl-H), 7.50 (d, *J* = 16.0 Hz, 1H, alkene-H), 7.56 (d, *J* = 8.0 Hz, 1H, phenyl-H), 7.73(s, 1H, phenyl-H), 7.87 (t, *J* = 8.0 Hz, 1H, phenyl-H), 7.92 (d, *J* = 8.0 Hz, 1H, phenyl-H), 8.00 (d, *J* = 8.0 Hz, 1H, phenyl-H), 8.04 (t, *J* = 8.0 Hz, 1H, phenyl-H), 8.24 (d, *J* = 8.0 Hz, 1H, phenyl-H), 8.90 (d, *J* = 8.0 Hz, 1H, phenyl-H). ^13^C NMR (100 MHz, DMSO-*d*_6_, ppm): δ 165.84, 151.86, 150.47, 150.34, 143.33, 138.18, 135.37, 132.98, 130.47, 128.51, 124.97, 124.23, 122.31, 121.25, 120.70, 120.40, 117.76, 104.59, 43.02, 17.53, 13.63. Mass spectrometry (ESI-MS, *m/z*): [M – H]^−^ calcd. for [C_30_H_22_N_4_OS – H]^−^ 485.1436; found 485.1439.

### Synthesis of QM-HBT-βgalAc

QM-HBT-OH (100 mg, 0.21 mmol) and tetra-O-acetyl-α-D-galactopyranosyl-1-bromide (150 mg, 0.36 mmol) were dissolved in acetonitrile (15 ml) with Cs_2_CO_3_ (359 mg, 1.10 mmol) and Na_2_SO_4_ (125 mg, 0.88 mmol) under argon protection at room temperature. The mixture then was stirred at room temperature for 4 h. After filtration, the solvent was removed under reduced pressure. The residue was taken up in sat.NH_4_Cl and extracted with CH_2_Cl_2_. Next, the solution was dried over anhydrous Na_2_SO_4_, and the solvent was removed by evaporation again. Finally, the crude product was purified by silica gel chromatography using dichloromethane/methanol (100:1) to afford the desired product QM-HBT-βgalAc as a red solid (30 mg): yield 18%. ^1^H NMR (400 MHz, DMSO-*d*_6_, ppm) δ 1.41 (t, *J* = 8.0 Hz, 3H, NCH_2_*C**H***_3_-H), 1.76 (s, 3H, acetyl-H), 1.94 (s, 3H, acetyl-H), 1.99 (s, 3H, acetyl-H), 2.08 (s, 3H, acetyl-H), 2.46 (s, 3H, C***H***_3_-H), 3.56 (m, 2H, *C**H***_2_OCOCH_3_), 4.06 (t, *J* = 8.0 Hz, 1H, galactose-H), 4.62 (q, *J* = 8.0 Hz, 2H, N*C**H***_2_CH_3_-H), 5.16 (s, 1H, galactose-H), 5.24 (s, 1H, galactose-H), 5.34 (s, 1H, galactose-H), 5.48 (m, 1H, galactose-H), 7.06 (s, 1H, phenyl-H), 7.47 (t, *J* = 8.0 Hz, 1H, phenyl-H), 7.54 (t, *J* = 8.0 Hz, 1H, phenyl-H), 7.60 (d, *J* = 16.0 Hz, 2H, alkene-H), 7.65 (d, *J* = 8.0 Hz, 1H, phenyl-H), 7.95 (t, *J* = 8.0 Hz, 1H, phenyl-H), 8.01(s, 1H, phenyl-H), 8.11 (m, 2H, phenyl-H), 8.19 (d, *J* = 8.0 Hz, 1H, phenyl-H), 8.37(s, 1H, phenyl-H), 8.95 (d, *J* = 8.0 Hz, 1H, phenyl-H). ^13^C NMR (100 MHz, DMSO-*d*_6_, ppm): δ 169.67, 169.45, 163.39, 167.27, 152.34, 151.67, 149.11, 138.43, 137.75, 135.94, 133.74, 132.40, 132.29, 129.09, 127.67, 126.03, 125.12, 122.78, 121.61, 120.61, 118.12, 107.28, 99.94, 91.56, 70.10, 69.77, 69.18, 68.22, 67.00, 64.11, 61.19, 60.60, 47.20, 43.84, 20.52, 20.11, 16.21, 13.69. Mass spectrometry (ESI-MS, *m/z*): [M + H]^+^ calcd. for [C_44_H_40_N_4_O_10_S + H]^+^ 817.2543; found 817.2548.

### Synthesis of QM-HBT-βgal

QM-HBT-βgalAc (50 mg, 0.06 mmol) was added to MeONa (70 mg, 1.3 mmol) in methanol (10 ml), and the mixture was stirred at room temperature for 4 h. Then, the reaction mixture was neutralized with Amberlite IR-120 plus (H^+^). After Amberlite IR-120 plus (H^+^) was filtered off, the solvent was removed by evaporation. Finally, the crude product was purified by silica gel chromatography using dichloromethane/methanol (40:1) to afford the desired product QM-HBT-βgal (16 mg): ^1^H NMR (400 MHz, DMSO-*d*_6_, ppm) δ 1.41 (t, *J* = 8.0 Hz, 3H, NCH_2_*C**H***_3_-H), 2.53 (s, 3H, C***H***_3_-H), 3.07-3.26 (m, 4H, -O***H***), 3.27(m, 2H, *-C**H***_2_OH), 3.28 (m, 2H, galactose-H), 3.61 (d, *J* = 8.0 Hz, 1H, galactose-H), 3.87 (d, *J* = 8.0 Hz, 1H, galactose-H), 4.62 (q, *J* = 8.0 Hz, 2H, N*C**H***_2_CH_3_-H), 4.77 (d, *J* = 8.0 Hz, 1H, galactose-H), 7.07 (s, 1H, phenyl-H), 7.44 (t, *J* = 8.0 Hz, 1H, phenyl-H), 7.52 (d, *J* = 8.0 Hz, 1H, phenyl-H), 7.58 (d, *J* = 16.0 Hz, 2H, phenyl-H), 7.64 (d, *J* = 8.0 Hz, 1H, phenyl-H), 7.95 (d, *J* = 8.0 Hz, 1H, phenyl-H), 7.97 (s, 1H, phenyl-H), 8.09 (d, *J* = 16.0 Hz, 1H, alkene-H), 8.11 (d, *J* = 8.0 Hz, 1H, phenyl-H), 8.12 (d, *J* = 16.0 Hz, 1H, alkene-H), 8.39 (d, *J* = 8.0 Hz, 1H, phenyl-H), 8.95 (d, *J* = 8.0 Hz, 1H, phenyl-H). ^13^C NMR (100 MHz, DMSO-*d*_6_, ppm): δ 163.97, 153.87, 152.25, 151.58, 149.12, 138.67, 137.72, 136.00, 133.68, 132.49, 132.05, 131.29, 128.18, 127.56, 125.08, 125.01, 124.95, 122.52, 121.71, 120.89, 120.58, 119.15, 118.07, 107.19, 104.47, 75.48, 73.11, 71.11, 67.66, 59.79, 47.10, 43.85, 16.81, 13.71. Mass spectrometry (ESI-MS, *m/z*): [M + H]^+^ calcd. for [C_36_H_32_N_4_O_6_S + H]^+^649.2121; found 649.2123.

## Results and Discussion

### Design and Synthesis

In our strategy, QM is employed as the AIE building block along with generating modifiable sites for functionalization, which could perform controllable NIR emission via tuning electron–donor ability (Shi et al., 2013; Shao et al., 2014, 2015; Wang M. et al., 2018). Particularly, neighboring HBT moiety is covalent attached as hydrophobic π-conjugated backbone, efficiently extending emission wavelength to the NIR region. Finally, a β-gal activatable unit is grafted at the position of phenolic hydroxyl group, blocking the ESIPT process and endowing the elaborated probe QM-HBT-βgal with moderate water solubility. In consequence, the molecular dissolved state in the aqueous system renders the probe almost non-fluorescent. After being specifically hydrolyzed by β-gal, hydrophobic QM-HBT-OH can be released and aggregated with a remarkable light-up AIE-active fluorescent signal, which could be well retained in the reaction site and emit strong fluorescence for long-term tracking endogenous β-gal activity. The synthetic route for the probe is depicted in the Materials and General Methods (Scheme S1), which was fully characterized by ^1^H and ^13^C NMR and high-resolution mass spectrometry (HRMS) (Figures S10–S24).

#### Aggregation-Induced Emission Properties of QM-HBT-βgal and QM-HBT-OH

**Figure 1:**
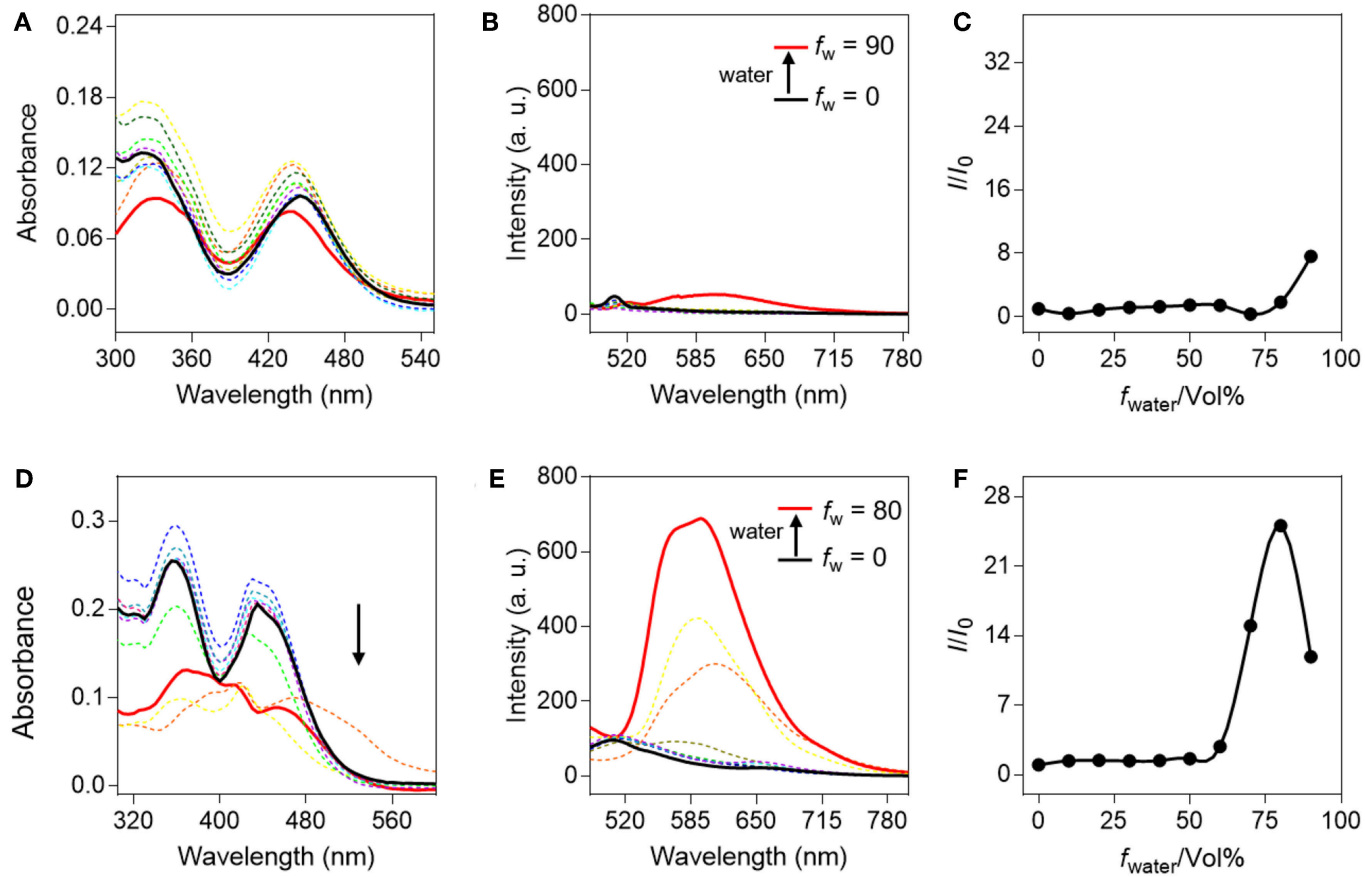
Spectral properties of QM-HBT-βgal and QM-HBT-OH with different water fractions (*f*_w_) in a mixture of water/tetrahydrofuran. **(A)** Absorption spectra, **(B)** emission spectra, and **(C)** *I*/*I*_0_ plots of QM-HBT-βgal (10 μM); **(D)** absorption spectra, **(E)** emission spectra, and **(F)** *I*/*I*_0_ plots of QM-HBT-OH (10 μM), where *I* is the fluorescence intensity at 620 nm and *I*_0_ is the fluorescence intensity of QM-HBT-βgal or QM-HBT-OH in 0% water, λ_ex_ = 460 nm.

Initially, the AIE properties of QM-HBT-βgal and QM-HBT-OH were investigated in the water/THF mixed solvents with different water volume fractions (*f*_w_ = 0–90%) in Figure 1. Actually, both compounds showed two absorption peaks at 360 and 440 nm, respectively. The absorption spectrum of QM-HBT-βgal displayed only slight changes with the increasing *f*_w_ (Figure 1A), but that of QM-HBT-OH significantly descended when the *f*_w_ exceeded 70% (Figure 1D), resulting from the light scattering effect of the well-formed nanoaggregates. As expected, the hydrophilic galactose group makes QM-HBT-βgal in the molecular dissolved state in water, which exhibits non-emission in various water/THF mixtures (Figures 1B,C), with a quantum yield (*Φ*) of 0.1 using Rhodamine B as a reference compound. In contrast, QM-HBT-OH showed distinct light-up AIE characteristics. By increasing the *f*_w_ from 0 to 60%, the emission intensity increased slowly, accompanied by an obvious bathochromic shift (Figure 1E). Further addition of water leads to a sharp and dramatic enhancement in fluorescence intensity (*Φ* = 3.6%), which correlates well with the formation of nanoaggregates in poorer solvents (Figure 1F). Here, the initial fluorescence*-off* state of QM-HBT-βgal and significant AIE-active NIR fluorescence of QM-HBT-OH in aggregated state made it as an ideal candidate for tracking β-gal.

#### Spectroscopic Properties and Optical Response to β-gal

**Figure 2:**
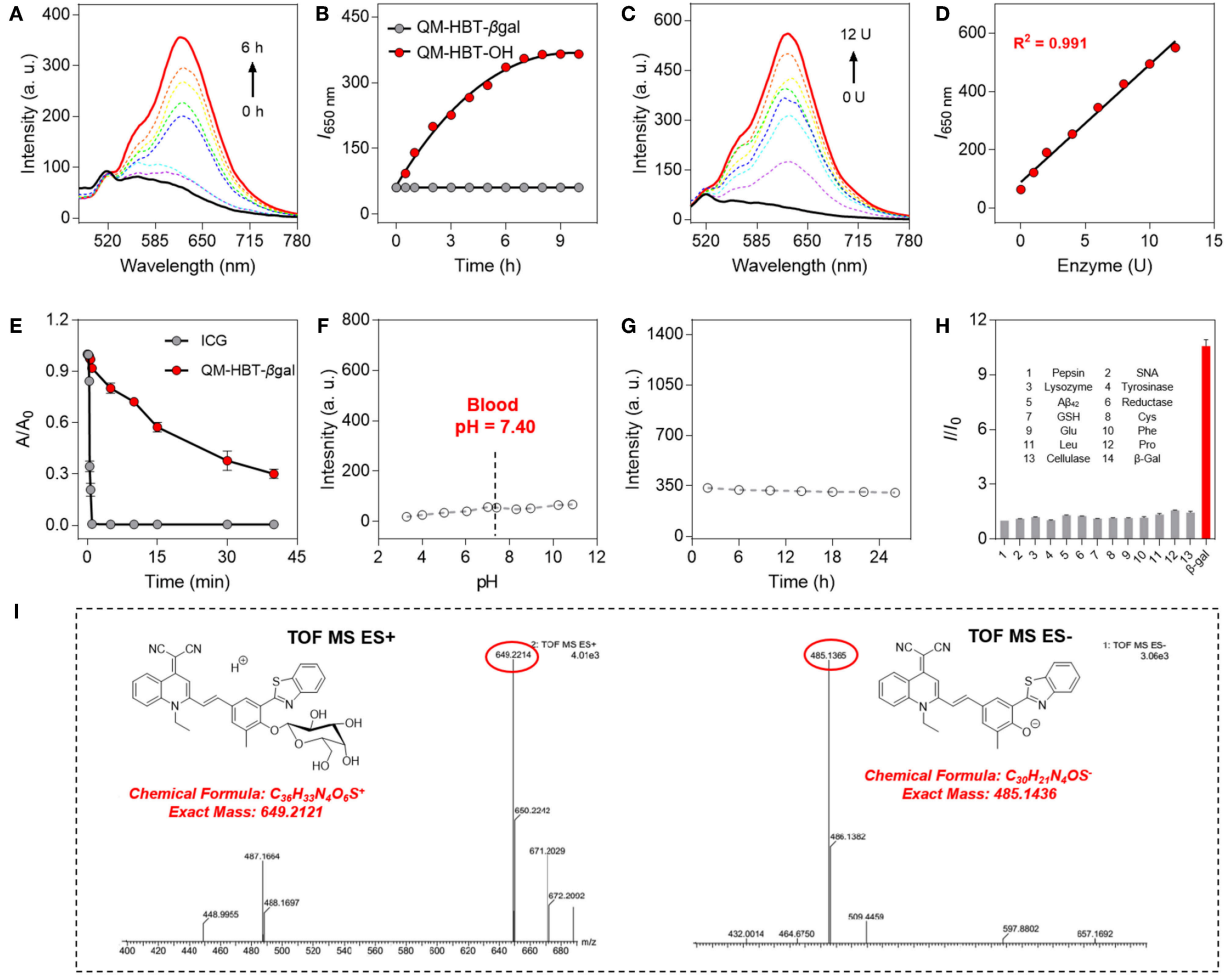
**(A)** Time-dependent fluorescence spectra of QM-HBT-βgal (10 μM) with 10 U β-gal in aqueous solution (PBS/DMSO = 7:3, v/v, 50 mM, pH = 7.4) at 37°C. **(B)** Time dependence of I_650_ nm for QM-HBT-βgal before (black) and after (red) adding β-gal, λ_ex_ = 460 nm. **(C)** Fluorescence spectra of QM-HBT-βgal (10 μM) upon treatment with increasing concentrations of β-gal (0–12 U) after incubation for 6 h. **(D)** Fluorescence intensity I_650_ nm as a function of β-gal concentration after incubation for 6 h. **(E)** Time-dependent fluorescence intensity of ICG (10 μM, monitored at 812 nm, and λ_ex_ = 780 nm) and QM-HBT-βgal (10 μM, monitored at 650 nm, and λ_ex_ = 460 nm) under sustained illumination. **(F)** Fluorescence intensity at 650 nm of QM-HBT-βgal remaining stable in various pH values and **(G)** fresh mouse serum over 24 h at 37°C. **(H)** Fluorescence responses of QM-HBT-βgal (10 μM) upon incubation with β-gal (10 U) and various other analytes (100 equiv.) in aqueous solution (PBS/DMSO = 7:3, v/v, 50 mM, pH = 7.4) at 37°C, λ_ex_ = 460 nm. **(I)** High-resolution mass spectrometry (HRMS) demonstrating the enzyme-activatable mechanism and showing the cleavage product QM-HBT-OH.

A smart AIE-active β-gal NIR fluorescent probe should significantly alter its fluorescence characteristic upon response to β-gal. With the probe QM-HBT-βgal in hand, we investigated the optical response of the probe to β-gal in the aqueous solution (PBS/DMSO = 7:3, v/v, 50 mM, pH = 7.4) at 37°C. QM-HBT-βgal has a broad absorption band at 440 nm, and the absorbance peak decreased gradually with the addition of β-gal (Figure S1). As shown in Figures 2A,B, a distinct NIR fluorescence enhancement at 650 nm was observed from QM-HBT-βgal upon the addition of 10 U β-gal, accompanied by a significant bathochromic shift in emission spectra. It could be interpreted that the QM-HBT-βgal can be specifically hydrolyzed by β-gal and then spontaneously form nanoaggregates QM-HBT-OH with a remarkable AIE-active fluorescent signal, which is confirmed by Figures S2, S3, and we observe *in situ* generation of about 200-nm nanoaggregates by DLS and SEM characterization. In addition, the enhancement of fluorescence intensity is dependent on the incubation time with β-gal, leveling off at around 6 h. In contrast, negligible fluorescence intensity change of QM-HBT-βgal was observed without the addition of β-gal, indicative of the moderate and efficient enzyme response rate toward β-gal. Further, we performed the titration to study the enzymatic fluorescence response of QM-HBT-βgal (Figure 2C). With the increase of β-gal concentration (0–12 U), the fluorescence intensity around 650 nm of QM-HBT-βgal gradually enhanced and showed a linear correlation vs. the concentration of β-gal in the range of 0–12 U with a correlation coefficient of 0.991 (Figure 2D). Obviously, these remarkable characteristics of QM-HBT-βgal indicated that it could be capable of detecting β-gal activity.

#### Photostability and Selectivity

The high photostability of QM-HBT-βgal is very crucial to perform long-term tracking and high-fidelity imaging of enzyme activity in preclinical applications. Compared with the commercial FDA-approved NIR contrast agent ICG, the time-dependent absorbance measurements were also conducted to evaluate the photostability of QM-HBT-βgal upon continuous illumination (Hamamatsu, LC8 Lightningcure, 300 W) in aqueous solution (Figure 2E). As calculated, the half-life of QM-HBT-βgal (~1,500 s) is 60-fold longer than that of ICG (~25 s), suggestive of excellent photostability of QM-HBT-βgal, which further confirmed its potential application in long-term tracking.

We further study the stability of the probe QM-HBT-βgal in different pH values. Figure 2F indicates excellent stability of QM-HBT-βgal in various pH values (3–11), and its excellent stability remained in fresh mouse serum over 24 h (Figure 2G). In addition, the probe QM-HBT-βgal exhibits excellent pH stability in the medium of FBS (Figures S4, S8 and S9). Furthermore, prior to investigating endogenous β-gal activity in living cells, the specificity of QM-HBT-βgal toward β-gal was also evaluated with potential competitive species including amino acids, enzymes, serum markers, and metabolic substances. As shown in Figure 2H, compared with β-gal, nearly negligible fluorescence change of QM-HBT-βgal makes it a promising candidate to achieve accurate detection under practical applications.

#### Sensing Mechanism

For gaining insight into the activation mechanism of probe QM-HBT-βgal for enzyme β-gal, and then *in situ* release of QM-HBT-OH as NIR AIEgens, we acquired high-resolution mass spectrometry to confirm this proposed mechanism. In the electrospray ionization (ESI)-MS spectra of QM-HBT-βgal with β-gal, the peaks of QM-HBT-βgal and QM-HBT-O^−^ were found at *m/z* 649.2214 and 485.1365 (Figure 2I), respectively. The result clearly indicated that QM-HBT-βgal could be specifically activated by enzyme β-gal, and release QM-HBT-OH as NIR AIEgens.

#### Imaging of Endogenous β-gal in Living Cells

In order to study the biocompatibility of probe QM-HBT-βgal, standard 5-diphenyltetrazolium bromide (MTT) assays in human ovarian carcinoma cells (SKOV-3 cells) and human epithelioid cervical carcinoma cells (Hela cells) were carried out, respectively. As is observed in Figure S5, experimental results verified that probe QM-HBT-βgal has almost no cytotoxicity toward living cells.

**Figure 3:**
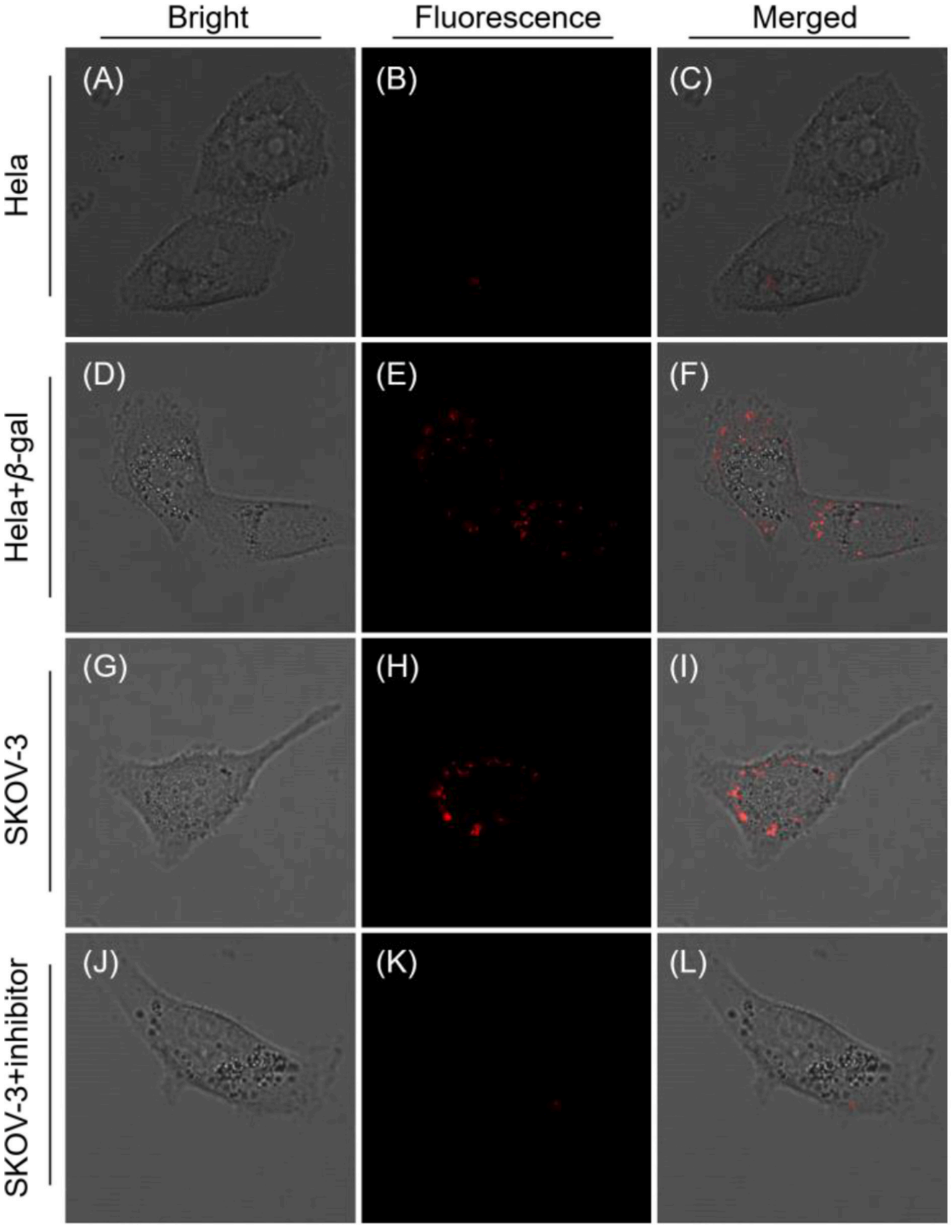
Confocal laser scanning microscopy (CLSM) images of Hela and SKOV-3 cells incubated with QM-HBT-βgal (10 μM) for 4 h: **(A–C)** Hela cells, **(D–F)** Hela cells pretreated with 1 mM β-gal for 0.5 h. **(G–I)** SKOV-3 cells, and **(J–L)** SKOV-3 cells pretreated with 1 mM inhibitor for 0.5 h. The window of fluorescence emission collection is 650–700 nm, λ_ex_ = 460 nm.

Taken all together, the probe QM-HBT-βgal is anticipated to be capable of accurately detecting the endogenous enzyme activity in living cells with *in situ* formation of AIEgen nanoaggregates. To demonstrate this potential, SKOV-3 cells were used as a model, because they overexpress β-gal (Asanuma et al., 2015), while Hela cells without expressed β-gal were used as a negative control model. As depicted in Figures 3A–C, no fluorescence was observed at the Hela cells, in accordance with the weak fluorescence spectrum of QM-HBT-βgal. In contrast, by adding an exogenous 10 U β-gal to Hela cells, a significant enhancement of fluorescence (Figures 3D–F) was observed due to further the enzyme conversion process. As demonstrated, the enzyme-catalyzed AIE-active NIR fluorescent probe QM-HBT-βgal can be used to detect β-gal in cancer cells. Impressively, SKOV-3 cells, which were treated with QM-HBT-βgal for 3 h, exhibit strong fluorescence in its NIR fluorescent channel (Figures 3G–I), suggesting its possible reaction with endogenous β-gal in the cells. These AIE-active signals were interpreted as being insoluble aggregates of released QM-HBT-OH, only occurring at the site where the probe is reacted with β-gal. To verify that the fluorescence change was caused by endogenous β-gal, 1 mM D-galactose (an inhibitor of β-gal; Portaccio et al., 1998) was used to pretreat the cells for 0.5 h. Negligible fluorescence signal was observed in the NIR channel (Figures 3J–L), indicating that the enhancement in SKOV-3 cells indeed results from the endogenous β-gal activity.

**Figure 4:**
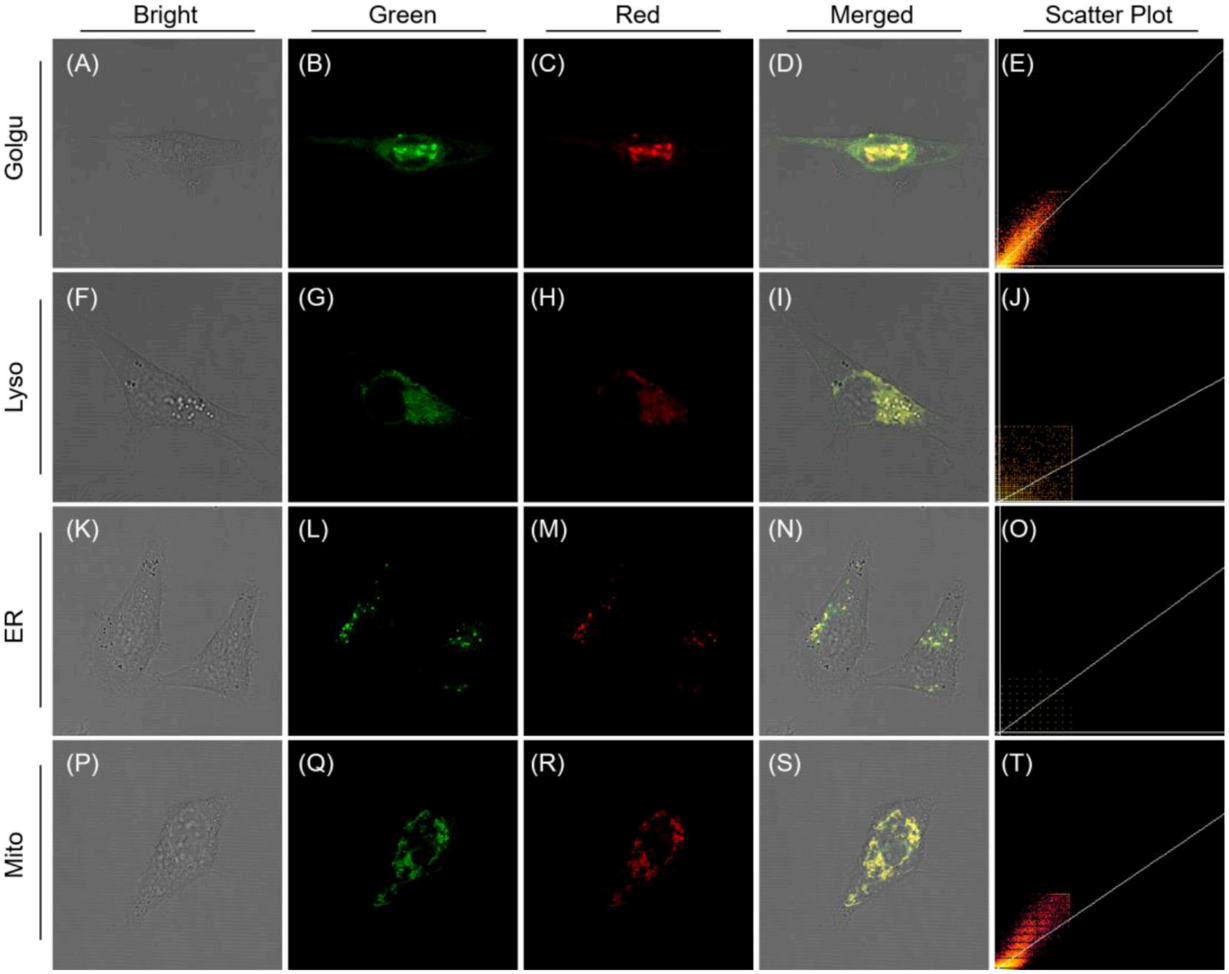
CLSM images for intracellular localization of QM-HBT-βgal in SKOV-3 cells. Cells were incubated with QM-HBT–βgal (10 μM) for 2 h and then co-stained with 1 μM Golgi-Tracker Green (BODIPY FL C5-Ceramide) **(A–E)**, 100 nM Lyso-Tracker Red DND-99 **(F–J)**, 1 μM ER-Tracker Red (BODIPY^®^ TR Glibenclamide) **(K–O)**, and 200 nM Mito-Tracker Red for 30 min **(P–T)**, respectively. The green channel at 510–530 nm for Golgi-Tracker Green **(B)**, λ_ex_ = 505 nm; 610–630 nm for ER-Tracker Red **(L)**, 590–610 nm for Lyso-Tracker Red DND-99 **(G)** and Mito-Tracker Red **(Q)**, λ_ex_ = 561 nm. The red channel at 650–700 nm, λ_ex_ = 460 nm.

To establish the precise intracellular localization, co-staining experiments of QM-HBT-βgal were performed with SKOV-3 cells. The AIE-active probe QM-HBT-βgal co-staining with commercially available Golgi-Tracker Green, LysoTracker Red, ER-Tracker Red, and Mito-Tracker Red shows an obvious co-localization characteristic (Figure 4). Specifically, the red channel from AIEgen nanoaggregates largely overlaps with the green channel from Mito-Tracker Red and Golgi-Tracker Red, with Pearson's correlation coefficients of 0.8977 and 0.8522 (Table S1). These results indicate that enzyme-catalyzed AIEgen QM-HBT-OH tends to mainly accumulate in the mitochondria and Golgi body.

**Figure 5:**
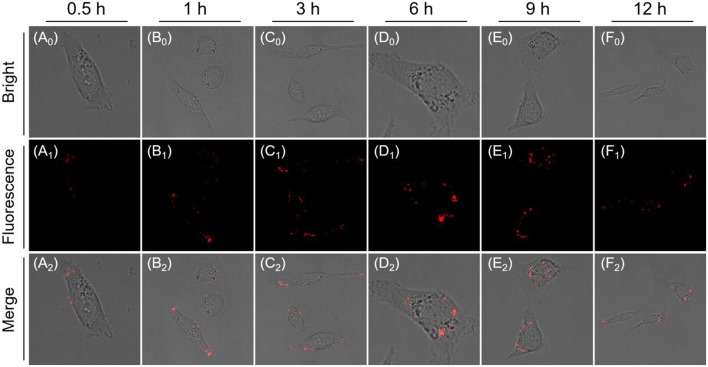
Time-dependent CLSM images of SKOV-3 cells incubated with QM-HBT-βgal (10 μM) (**A**_**0**_**-F**_**0**_: Bright field, **A**_**1**_**-F**_**1**_: Fluorescence channel) and (**A**_**2**_**-F**_**2**_: Merge channel). The window of fluorescence emission collection is 650–700 nm (λ_ex_ = 460 nm).

Furthermore, long-term tracking of endogenous β-gal experiments also conducted in living cells. Indeed, its fluorescence intensity slowly increases and reaches a plateau at 3 h, which does not change significantly over the next 3 h (*t* = 6 h; Figure 5), suggesting the AIE-active NIR fluorescent signal is ascribed to nanoaggregate formation, which could amplify the fidelity signals because of emitting stronger during concentration enrichment of AIEgen nanoaggregates. Most importantly, AIEgen nanoaggregates do not easily leak out of cells during prolonged incubation, due to its lipophilicity of extending π-conjugated backbone. When the incubation time was increased to 12 h, the intracellular fluorescence intensity was slightly attenuated and was fixed in the local region (Figure 5). In addition, we use the commercial non-*in situ* fluorescent probe (ICG) and the reported probe DCM-βgal by introducing ACQ fluorophore dicyanomethylene-4*H*-pyran (DCM) as control compounds (Figures S6, S7), demonstrating the long-term tracking capability of NIR AIE-active QM-HBT-βgal.

Altogether, all these experiment results verified that the AIE-active NIR probe QM-HBT-βgal can overcome intracellular diffusion and attain high-fidelity enzyme information, enabling *in situ* and long-term tracking of β-gal in SKOV-3 cells.

## Conclusions

In summary, we have developed an enzyme-responsive NIR AIE-active probe QM-HBT-βgal for *in situ* and long-term tracking of endogenous β-gal activity, overcoming the dilemma of the requirement for released molecular fluorophores between diffusion-resistant and ACQ effect. Notably, QM-HBT-βgal is almost non-emissive in aqueous media, and upon the addition of β-gal, the masking groups at the hydroxyl moieties of QM-HBT-βgal were removed, thus recovering ESIPT and strong AIE-active NIR fluorescent signal in the aggregate states. Compared with other available β-gal probes, the AIE-active NIR probe QM-HBT-βgal not only provides localization signal of the β-gal at the reaction site but also avoids self-quenching when accumulated in cells, making obvious the advance in high-fidelity detection of endogenous enzyme activity. This study provides a promising strategy for the design of NIR AIE-active probes, paving a new pathway for *in situ* and long-term tracking of enzyme activity in preclinical applications.

## Author Contributions

WF and CY contributed equally. WF and CY was responsible for performing the experiments and writing manuscript. YZ and YM were responsible for providing cells. W-HZ were responsible for discussing experimental results. ZG was responsible for designing experiments and revising the paper.

### Conflict of Interest Statement

The authors declare that the research was conducted in the absence of any commercial or financial relationships that could be construed as a potential conflict of interest.
