# Supplementary material for: Near-Infrared Aggregation-Induced Emission-Active Probe Enables in situ and Long-Term Tracking of Endogenous β-Galactosidase Activity
Source: Front Chem. 2019 May 14;7:291. doi: 10.3389/fchem.2019.00291 (PMC6527754; doi:10.3389/fchem.2019.00291)
Supplement: Supplementary file 1 [file Data_Sheet_1.docx]

Supplementary Material

**Near-Infrared AIE-Active Probe Enable in Situ and Long-Term Tracking of Endogenous *β*-Galactosidase Activity**

**Wei Fu^§^, Chenxu Yan^§^, Yutao Zhang, Yiyu Ma, Zhiqian Guo*, and Wei-Hong Zhu**

*State Key Laboratory of Bioreactor Engineering, Shanghai Key Laboratory of Functional Materials Chemistry, Institute of Fine Chemicals, School of Chemistry and Molecular Engineering, East China University of Science & Technology, Shanghai 200237 China*

*E-mail: guozq@ecust.edu.cn*

**Table of Contents**

Page

**Scheme S1.** Synthesis of QM-HBT-OH, QM-HBT-*β*galAc and QM-HBT-*β*gal. S-3

**Table S1.** Pearson’s correlation coefficient in co-localization experiments. S-4

**Figure S1.** Absorption spectra of QM-HBT-*β*gal. S-5

**Figure S2.** Hydrodynamic diameter of QM-HBT-OH aggregates. S-5

**Figure S3.** SEM image of QM-HBT-OH aggregates. S-6

**Figure S4.** pH stability of QM-HBT-*β*gal in medium of fetal bovine serum (FBS). S-6

**Figure S5**. In vitro cytotoxicity of QM- HBT-*β*gal. S-7

**Figure S6.** Long-term tracking of *β*-gal in SKOV-3 cells using commercial ICG dyes. S-8

**Figure S7.** Long-term tracking of *β*-gal in SKOV-3 cells using ACQ probe DCM-*β*gal. S8-S9

**Figure S8**. Normalized fluorescence spectra of QM-HBT-OH in different solvents. S-9

**Figure S9.** Fluorescence spectra of QM-HBT-OH in different pH values. S-10

**Figure S10-S24.** Characterization of Intermediate Compound 1, 2, QM-HBT-OH, QM-HBT-*β*galAc and QM-HBT-*β*gal. S11- S18

1. Synthesis of QM-HBT-OH, QM-HBT-*β*galAc and QM-HBT-*β*gal


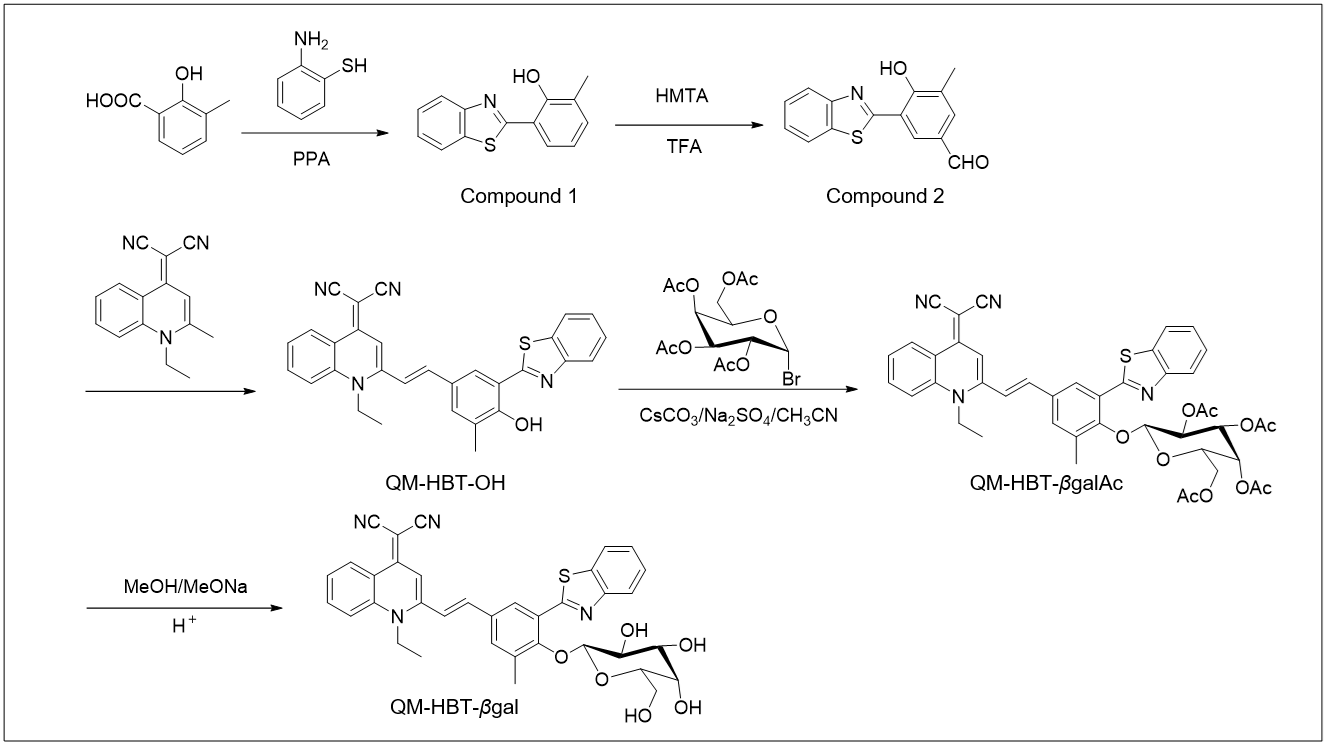


**Scheme S1.** Synthetic route of QM-HBT-OH, QM-HBT-*β*galAc and QM-HBT-*β*gal

1. Pearson’s correlation coefficient in co-localization experiments

**Table S1 Pearson’s correlation coefficient of various organelles in co-localization experiments**

| Organelle | Golgi body | Lysosome | Endoplasmic reticulum | Mitochondria |
| --- | --- | --- | --- | --- |
| Pearson’s correlation coefficient | 0.8522 | 0.5936 | 0.1829 | 0.8977 |

1. Absorption spectra of QM-HBT-*β*gal

Figure S1. Time-dependent absorption spectra of QM-HBT-*β*gal (10 μM) with 10 U *β*-gal in aqueous solution (PBS/DMSO = 7 : 3, v/v, 50 mM, pH = 7.4) at 37 C.

1. Hydrodynamic diameter of QM-HBT-OH aggregates


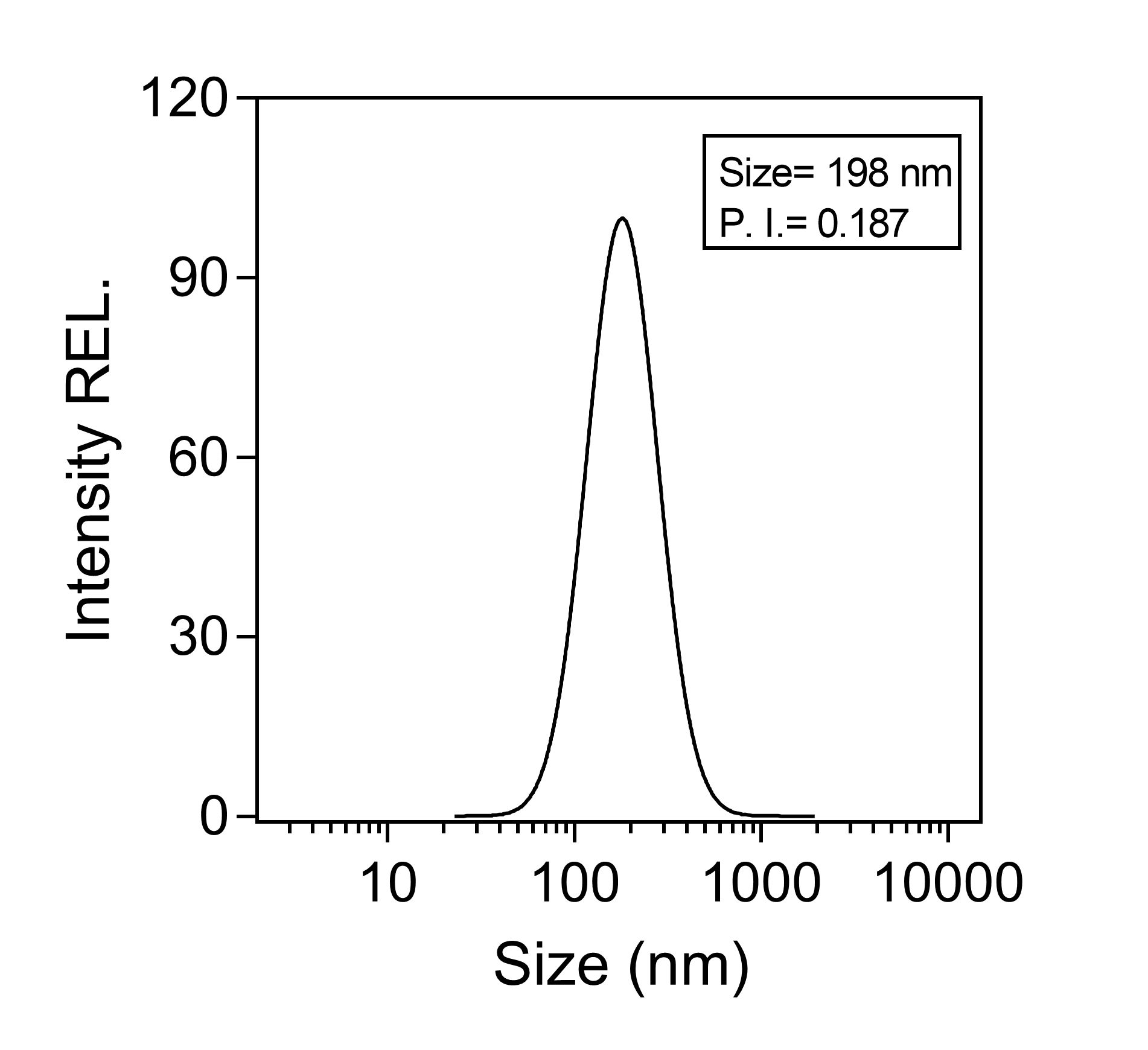


**Figure S2.** Hydrodynamic diameter of QM-HBT-OH (10 μM) in water obtained from dynamic light scattering (DLS).

1. SEM image of QM-HBT-OH aggregates


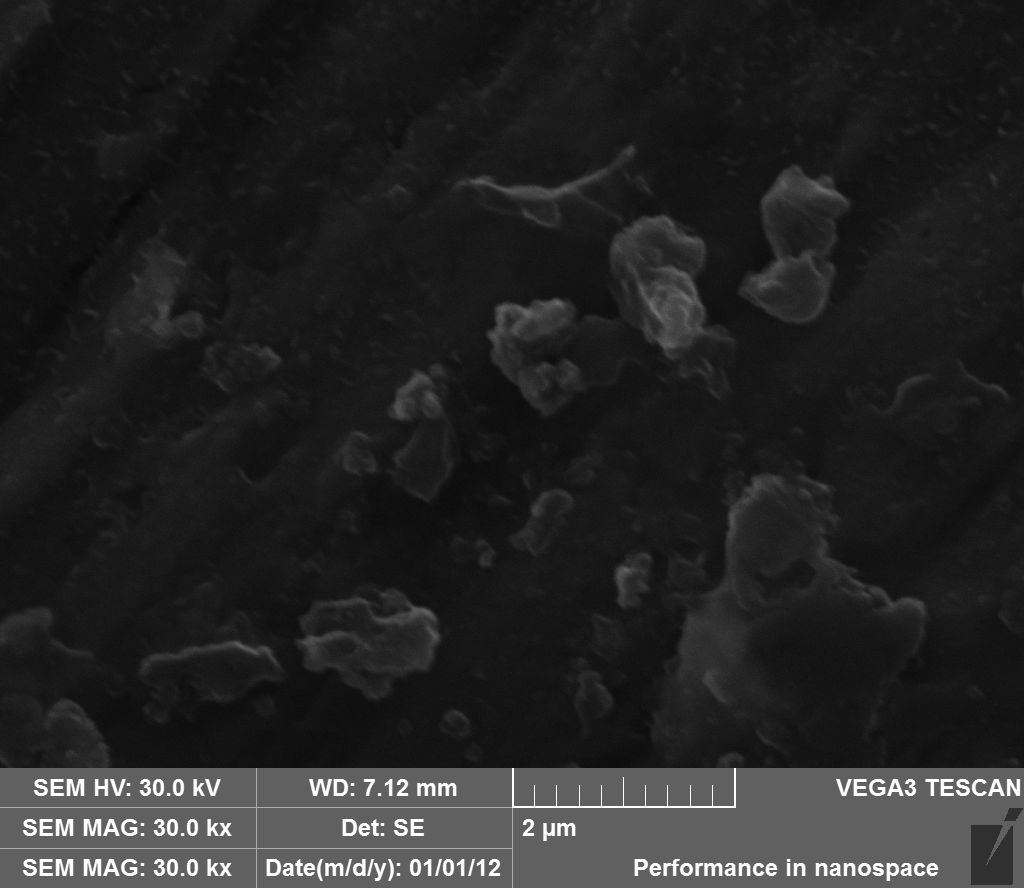


**Figure S3.** SEM images of nanoaggregates formed by QM-HBT-OH (10 μM) in water.

1. pH stability of QM-HBT-*β*gal in medium of fetal bovine serum (FBS)

Figure S4. Fluorescence intensity at 650 nm of QM-HBT-*β*gal in in medium of fetal bovine serum (FBS) remaining stable in various pH values.

1. In vitro cytotoxicity of QM- HBT-*β*gal

**Figure S5.** Cell viability after incubation of QM-HBT-*β*gal at different concentrations with the human ovarian adenocarcinoma cell line SKOV-3 cell line (Left) and human epithelioid cervical carcinoma HeLa cell line (Right) by MTT assay, at 37 °C for 24 h (each sample was tested using five replicates, and the results are reported as the mean ± standard deviation).

1. Long-term tracking of *β*-gal in SKOV-3 cells using commercial ICG dyes


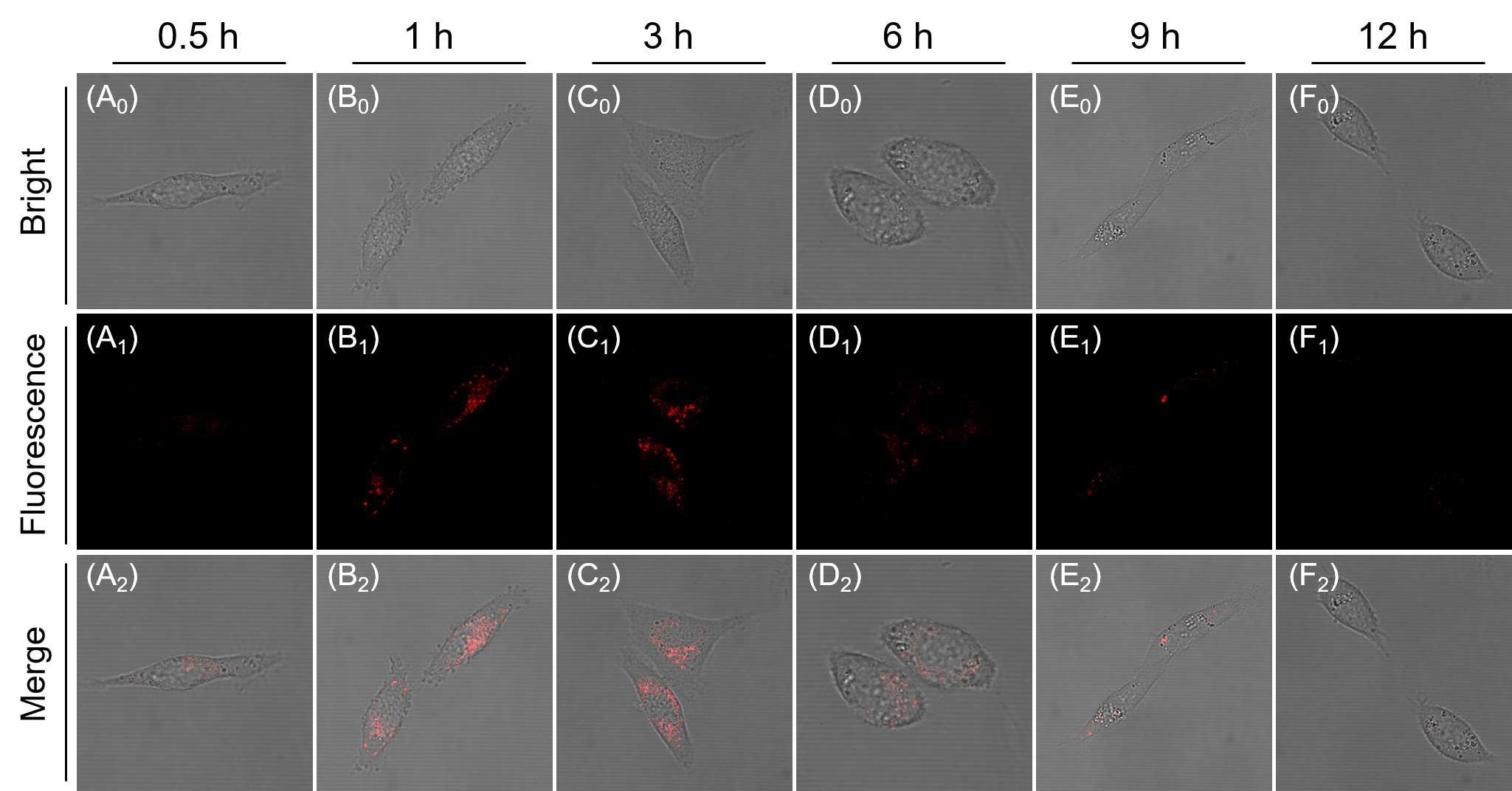


Figure S6. Time-dependent CLSM images of SKOV-3 cells incubated with commercial ICG (10 µM) (A_0_-F_0_: Bright field, A_1_-F_1_: Fluorescence channel) and (A_2_-F_2_: Merge channel). Fluorescence emission of ICG is 750-800 nm (*λ*_ex_ = 750 nm).

1. Long-term tracking of *β*-gal in SKOV-3 cells using ACQ probe DCM-*β*gal


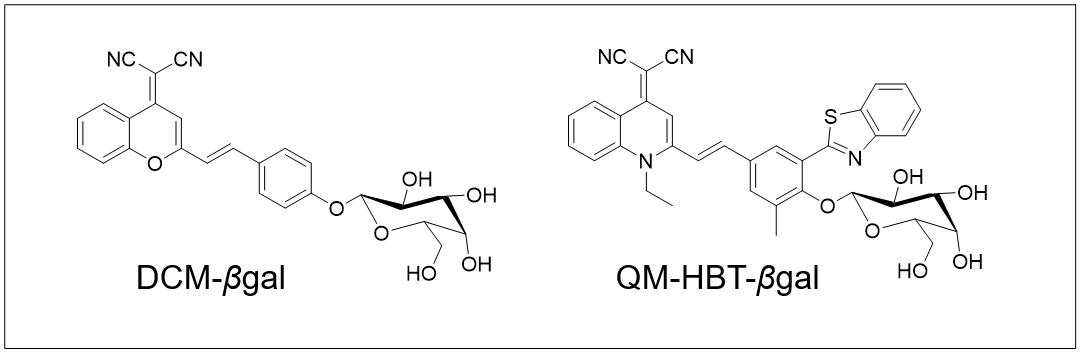


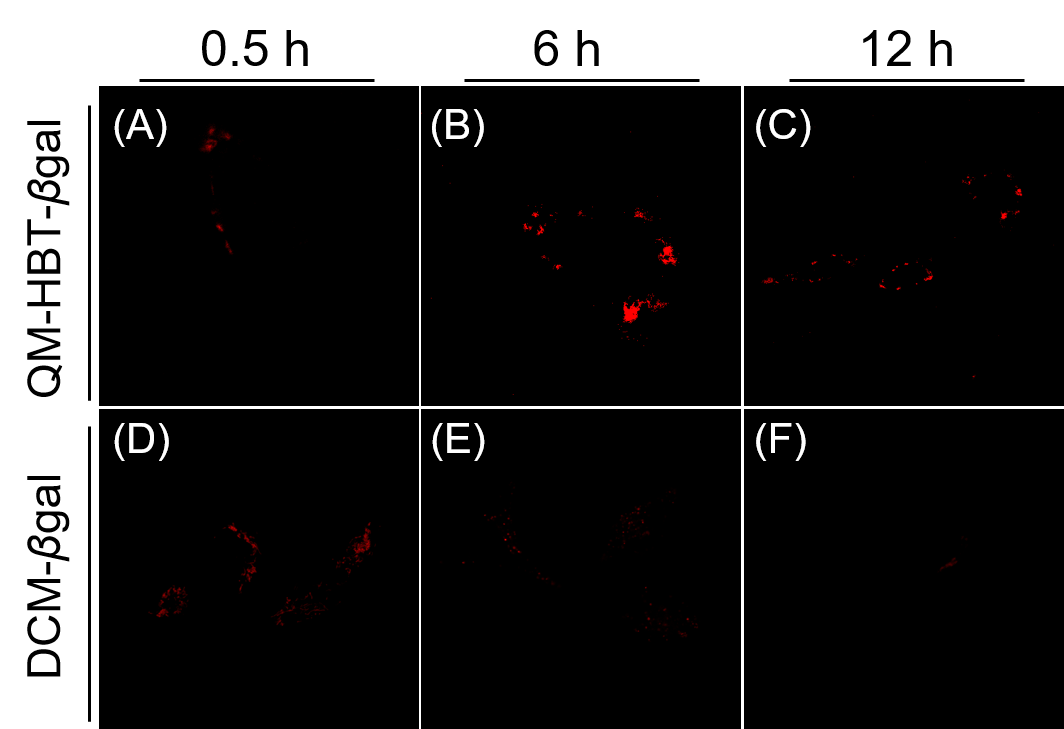


**Figure S7.** Time-dependent CLSM images of SKOV-3 cells incubated with (A-C) QM-HBT-*β*gal (10 µM) and (D-F) DCM-*β*gal (10 µM). Fluorescence emission of QM-HBT-*β*gal and DCM-*β*gal is 650-700 nm (*λ*_ex_ = 460 nm) and 605-725 nm (*λ*_ex_ = 560 nm), respectively.

1. Normalized fluorescence spectra of QM-HBT-OH in different solvents.

Figure S8. Normalized fluorescence spectra of QM-HBT-OH (10 μM) in different solvents.

1. Fluorescence spectra of QM-HBT-OH in different pH values.


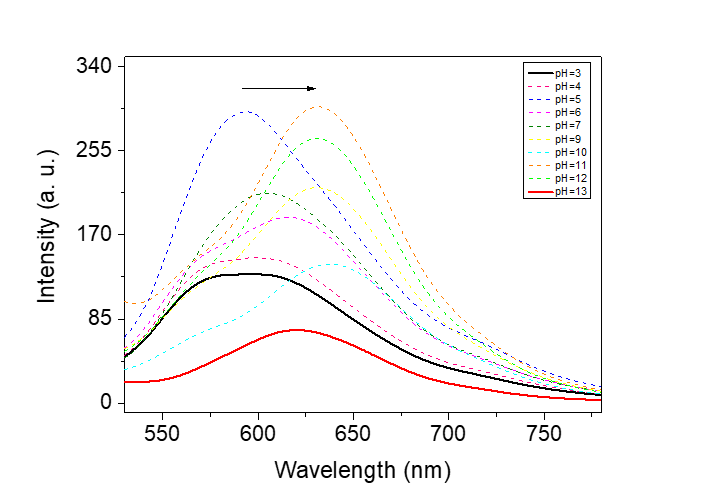


Figure S9. Fluorescence spectra of QM-HBT-OH (10 μM) in different pH values.

1. Characterization of Intermediate Compound 1, 2, QM-HBT-OH, QM-HBT-*β*galAc and QM-HBT-*β*gal


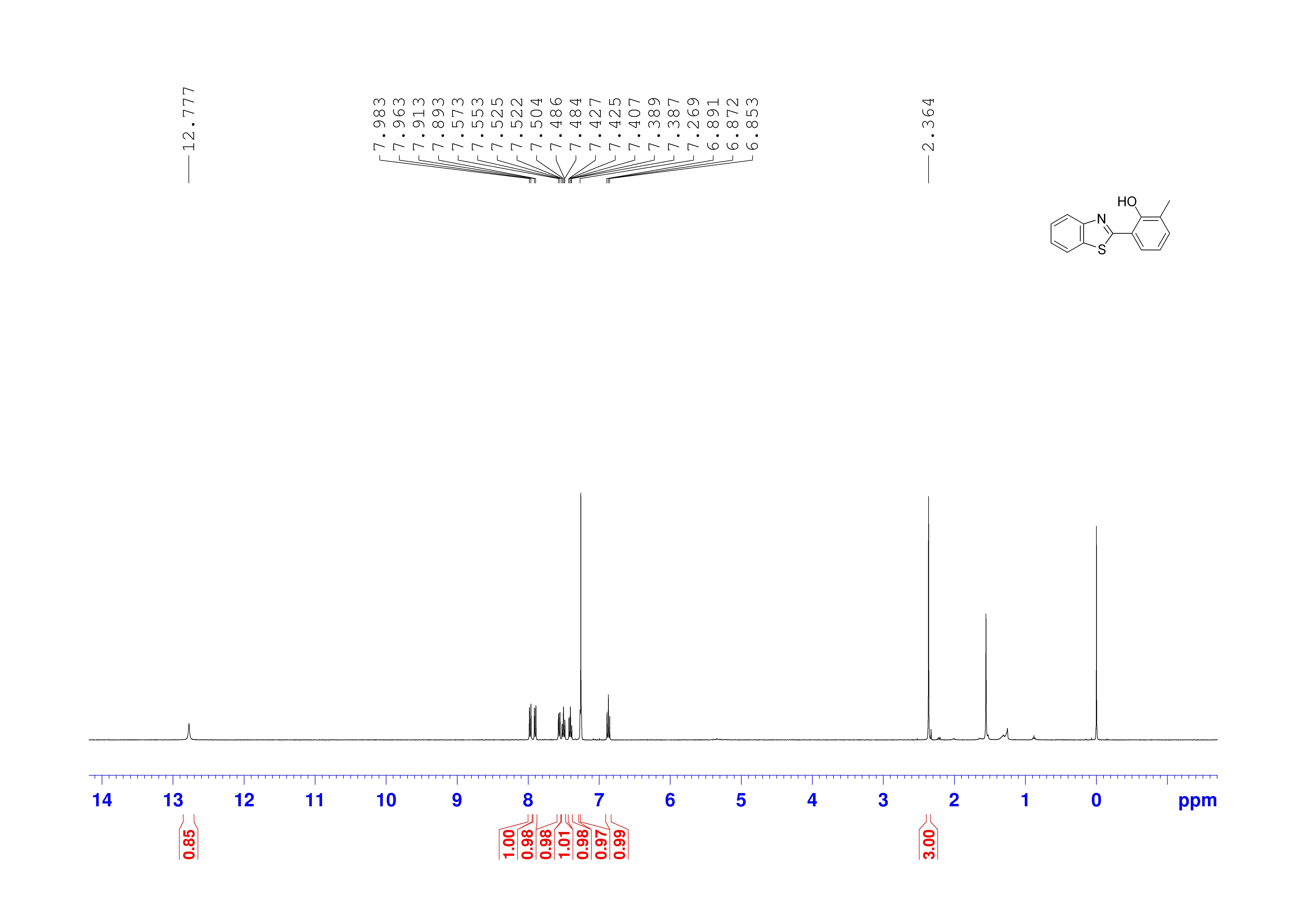


**Figure S10.** ^1^H NMR spectrum of compound 1 in CDCl_3_


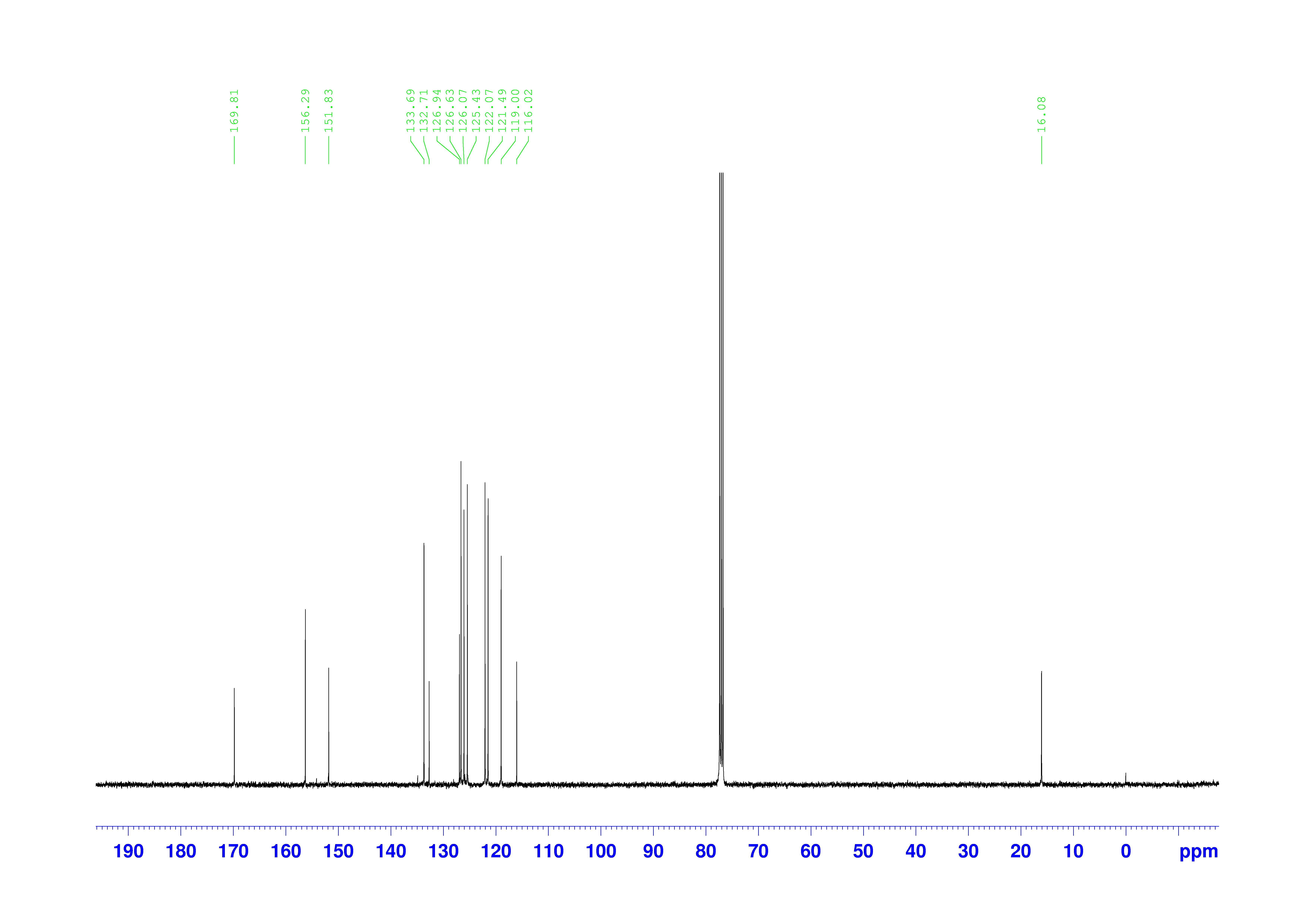


**Figure S11.** ^13^C NMR spectrum of compound 1 in CDCl_3_


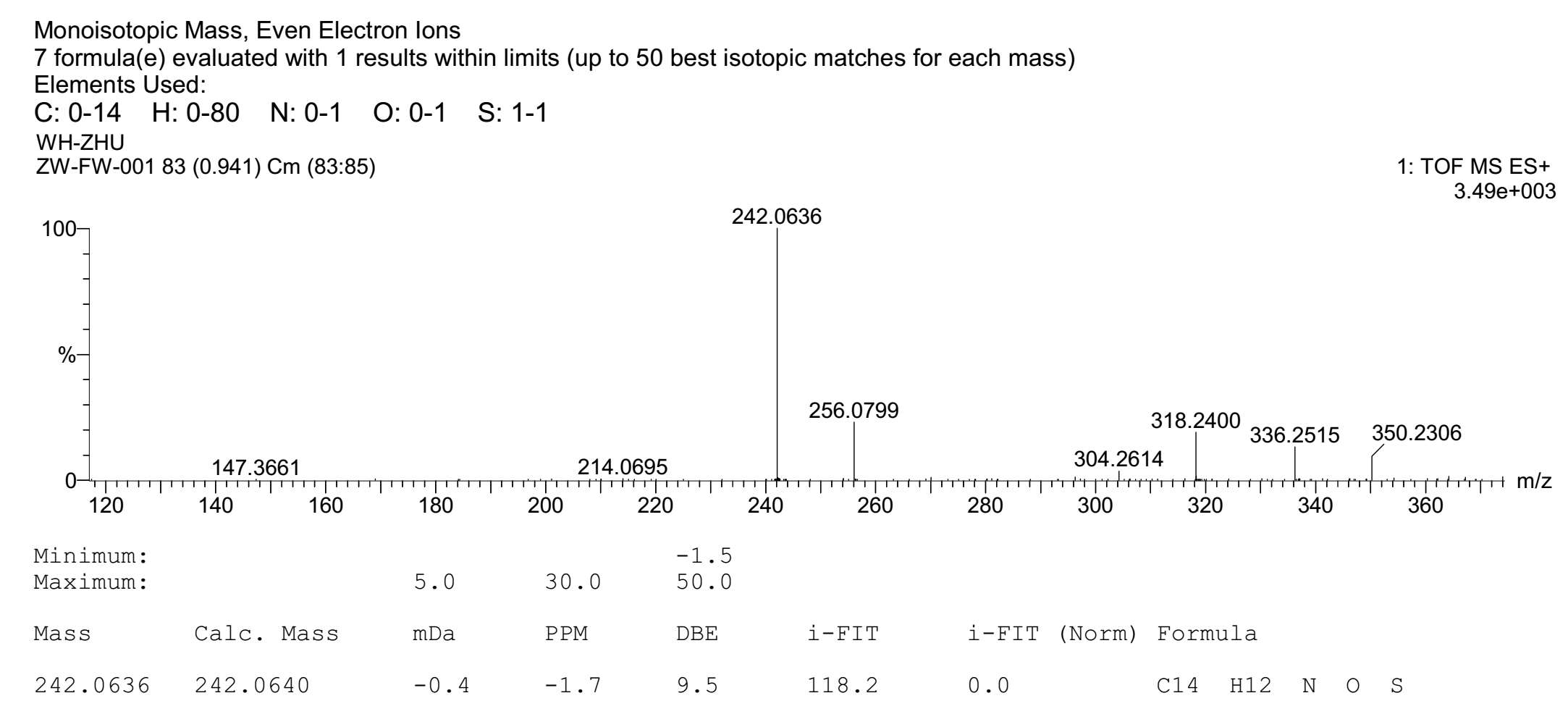


**Figure S12.** HRMS spectrum of compound 1.


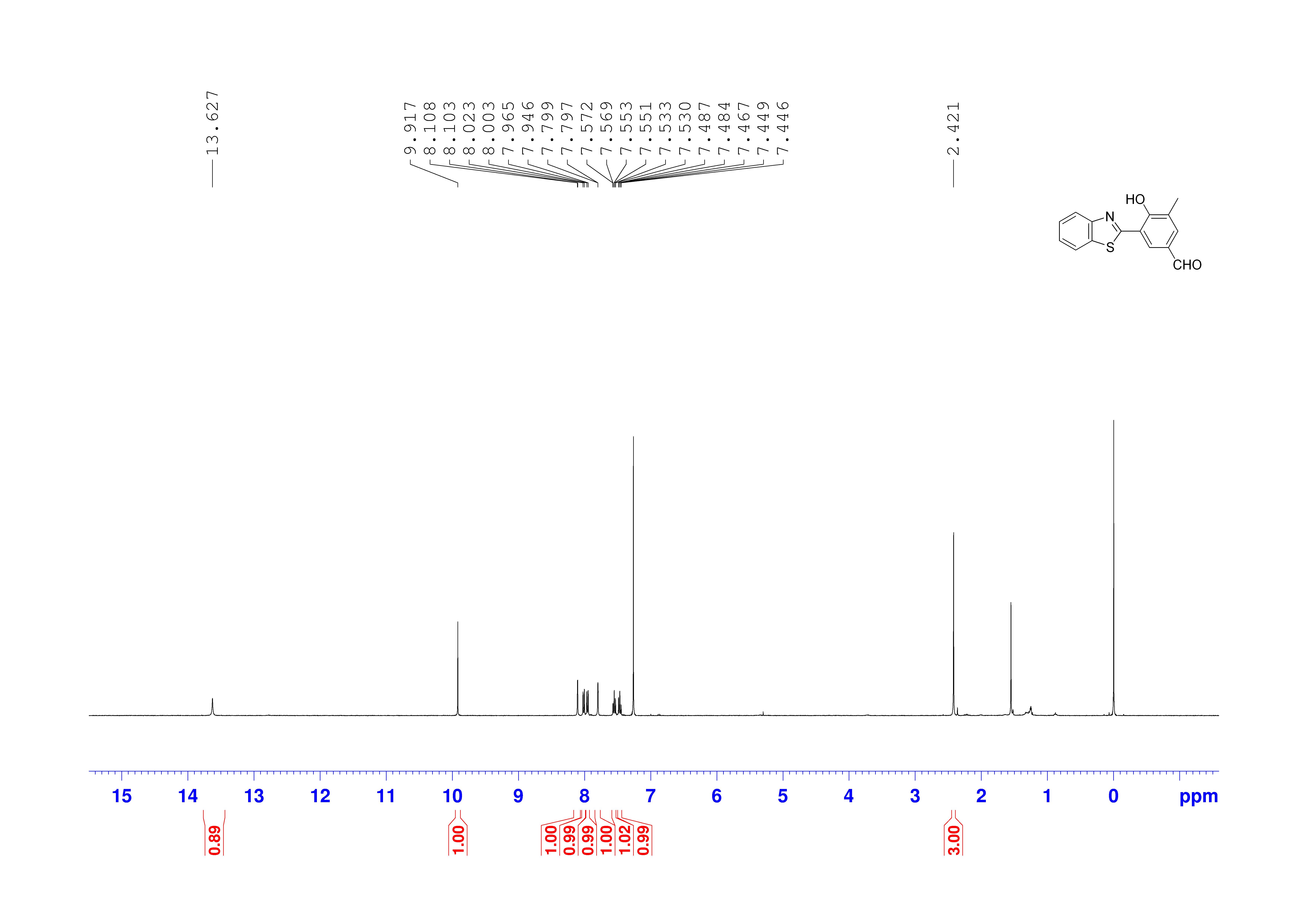


**Figure S13.** ^1^H NMR spectrum of compound 2 in CDCl_3_

_
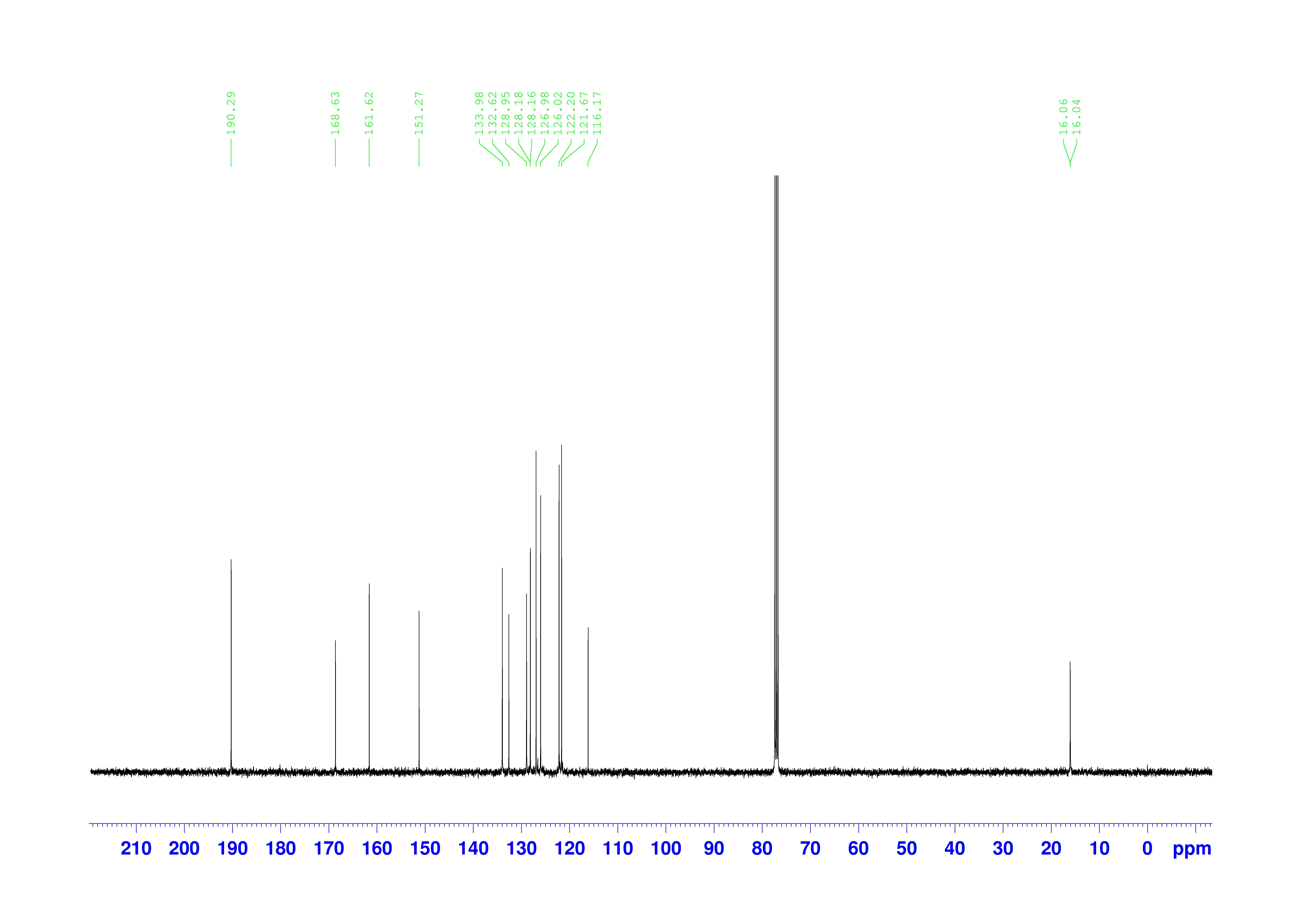
_

**Figure S14.** ^13^C NMR spectrum of compound 2 in CDCl_3_


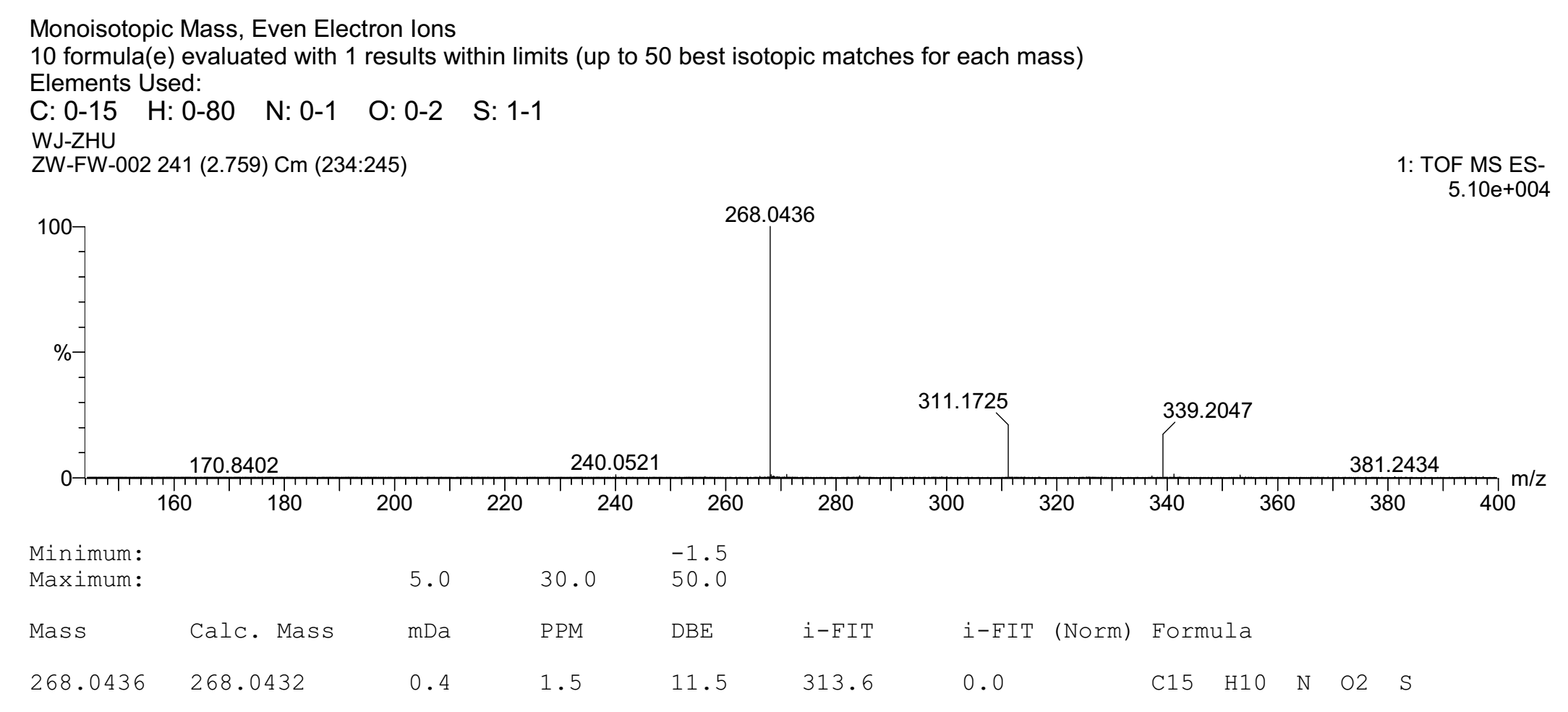


**Figure S15.** HRMS spectrum of compound 2.


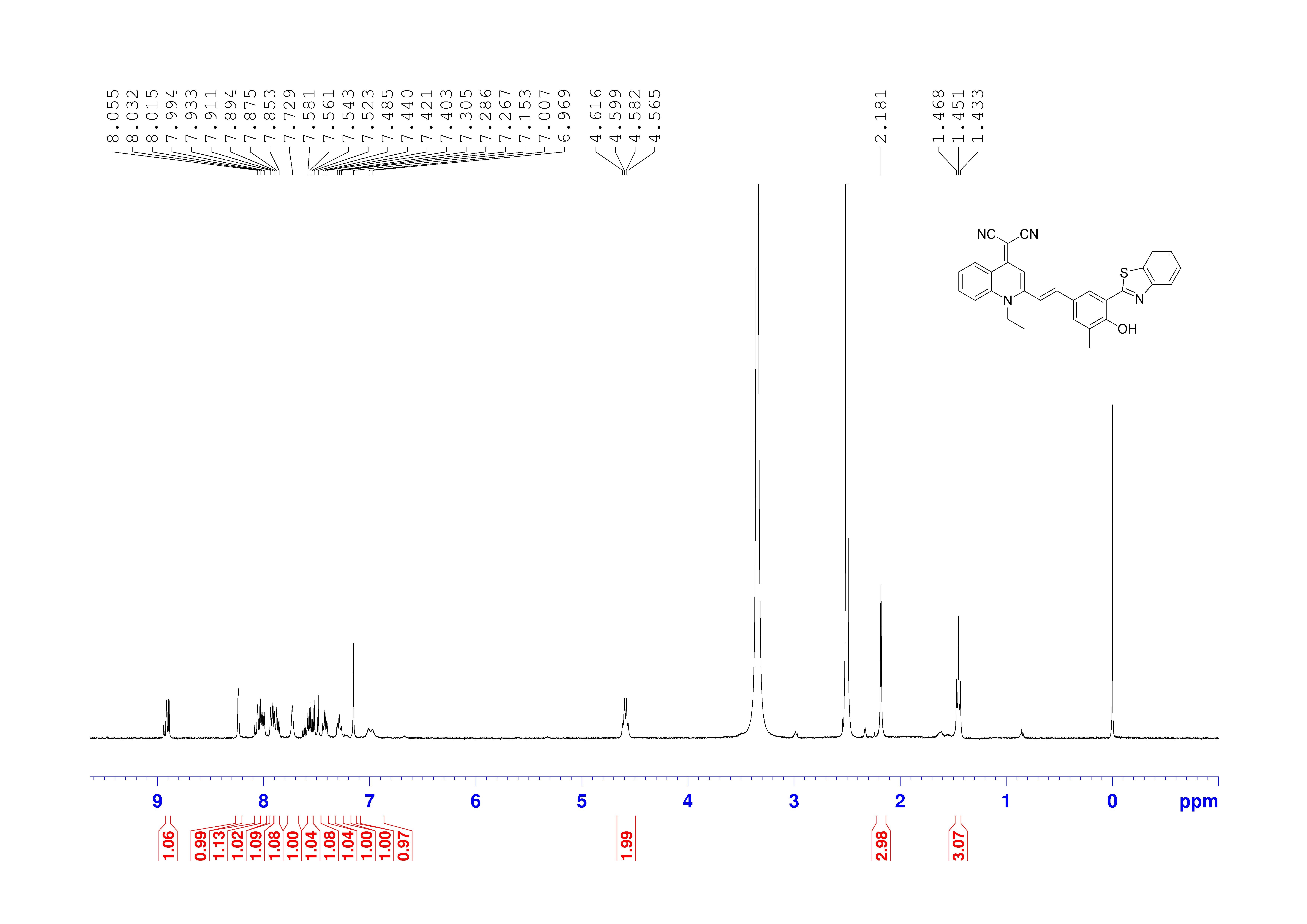


**Figure S16.** ^1^H NMR spectrum of QM-HBT-OH in DMSO-*d*_6_

_
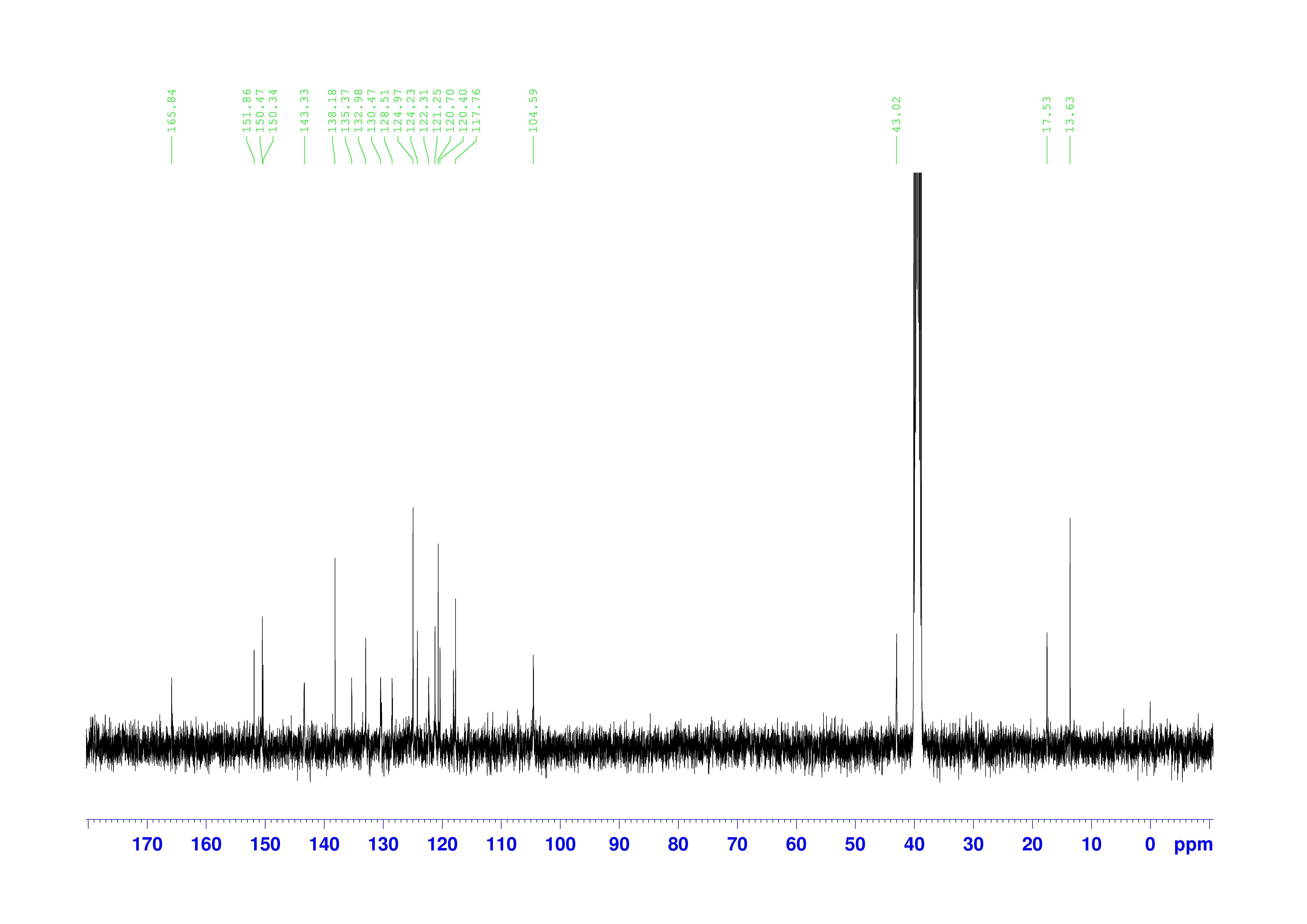
_

**Figure S17.** ^13^C NMR spectrum of QM-HBT-OH in DMSO-*d*_6_


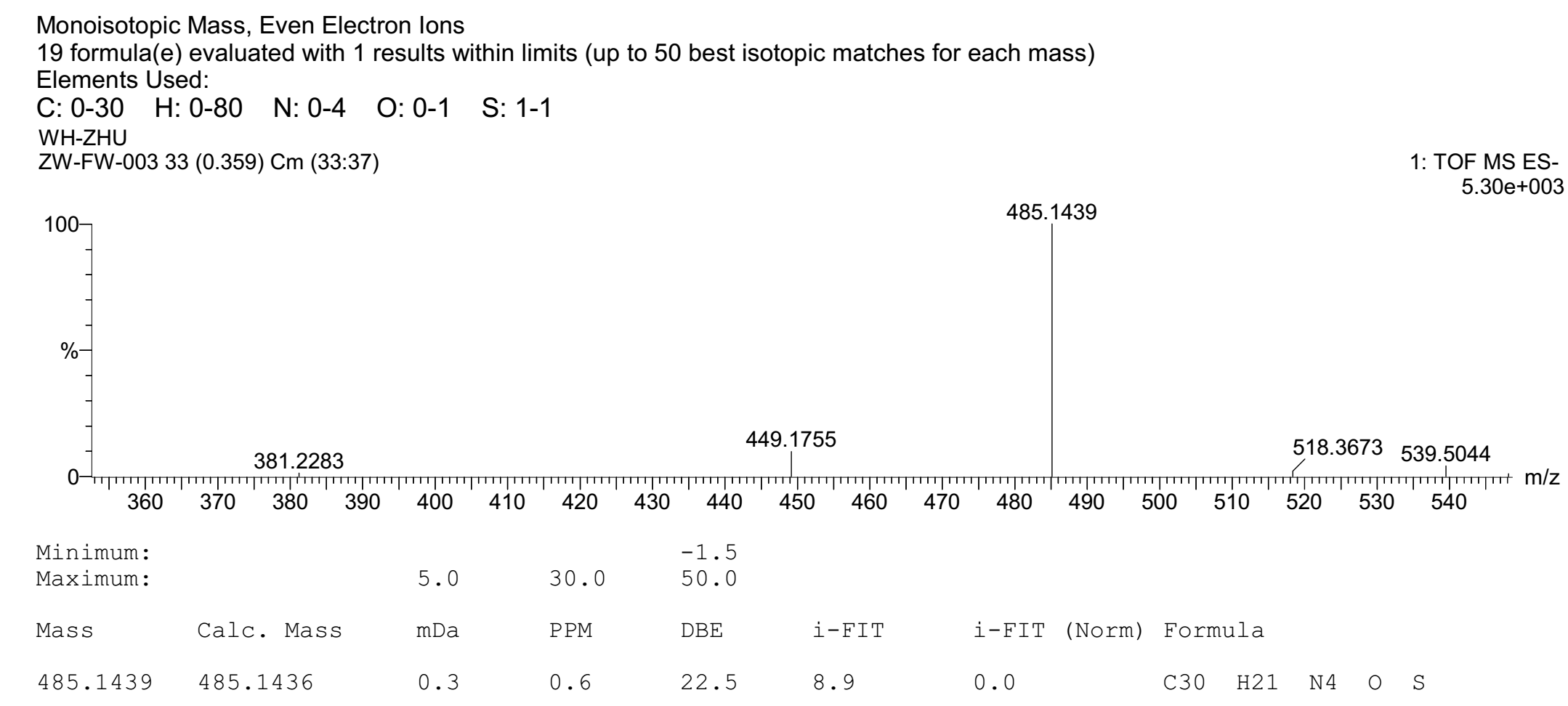


**Figure S18.** HRMS spectrum of QM-HBT-OH.


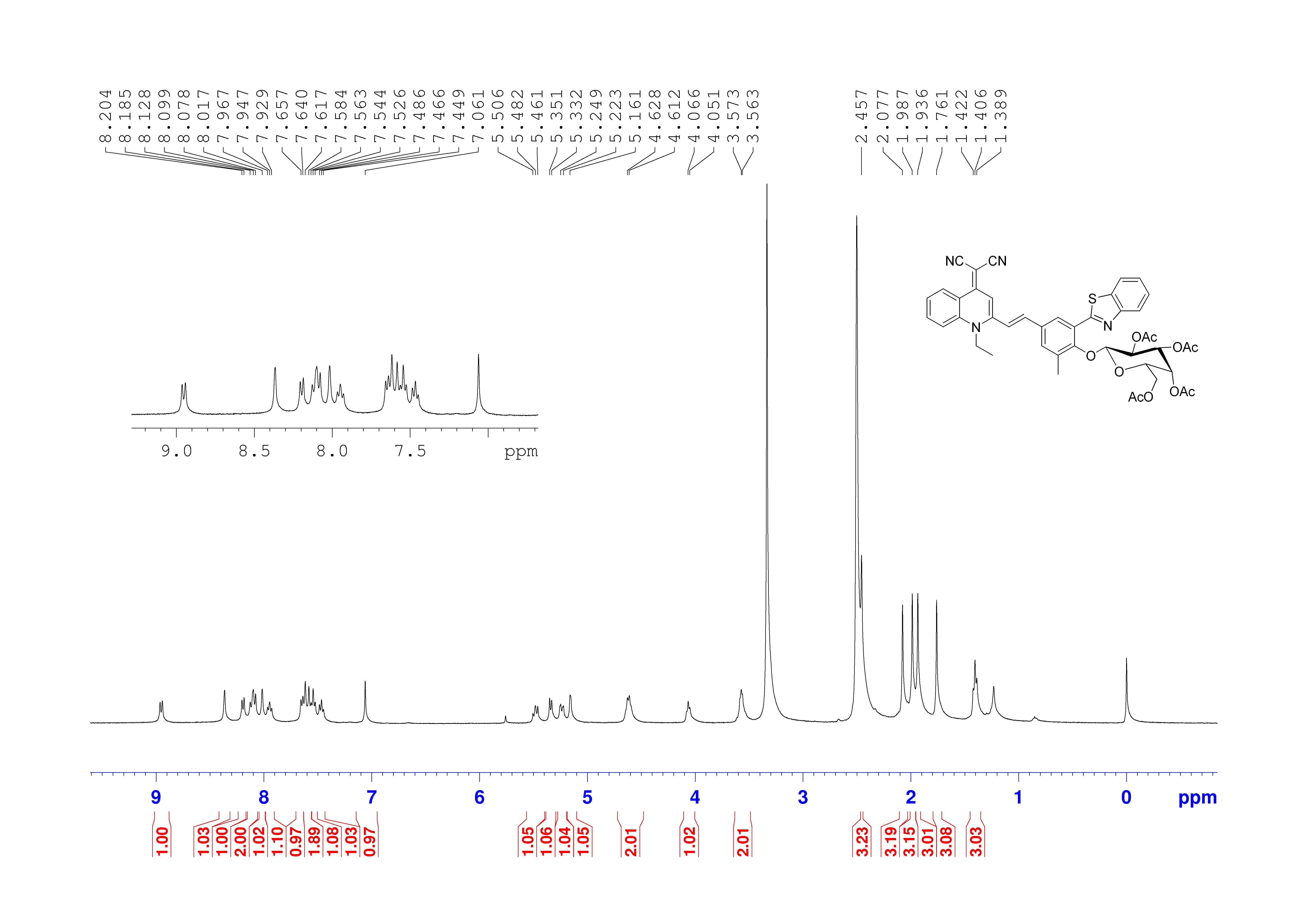


**Figure S19.** ^1^H NMR spectrum of QM-HBT-*β*galAc DMSO-*d*_6_

_
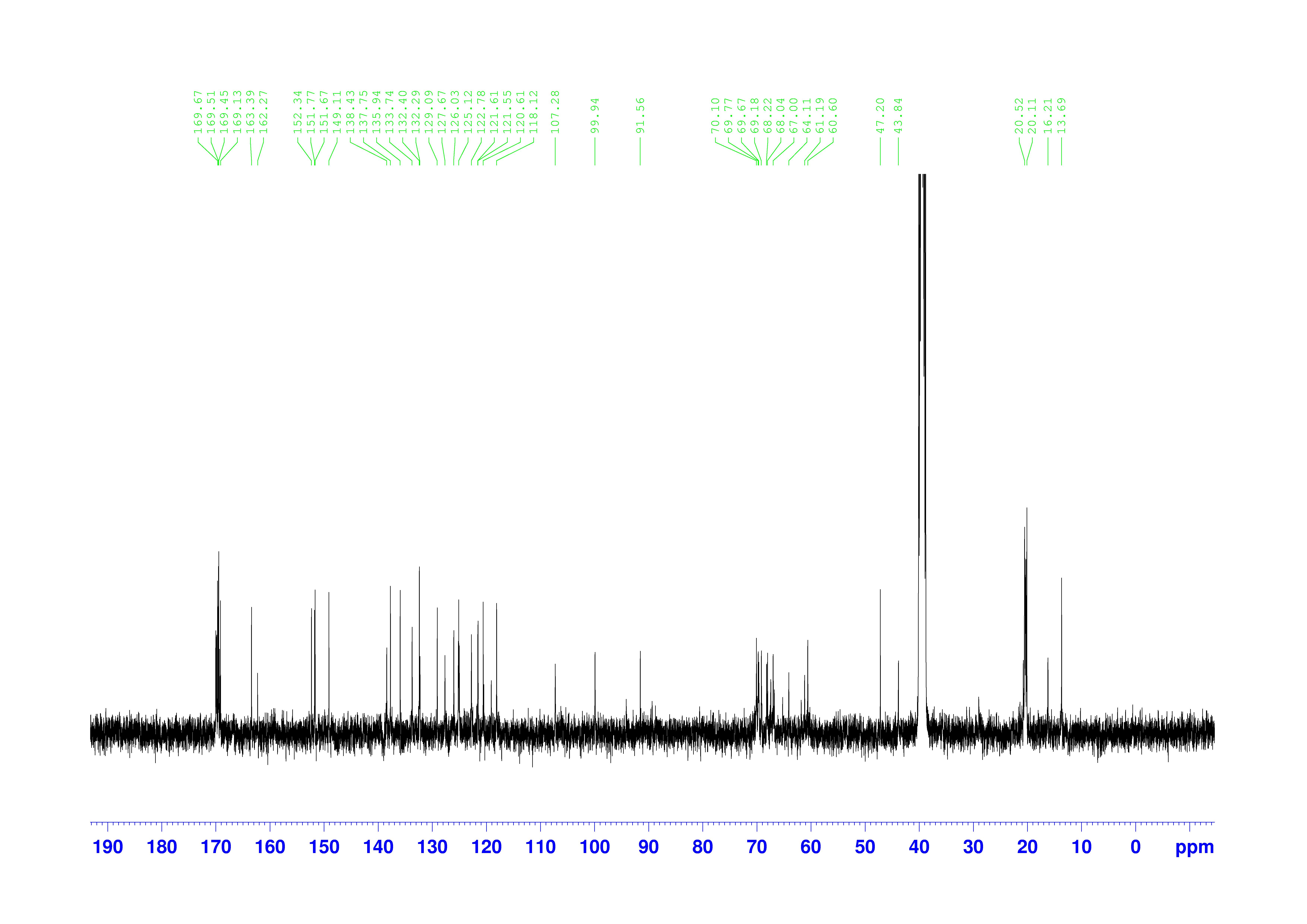
_

**Figure S20.** ^13^C NMR spectrum of QM-HBT-*β*galAc in DMSO-*d*_6_


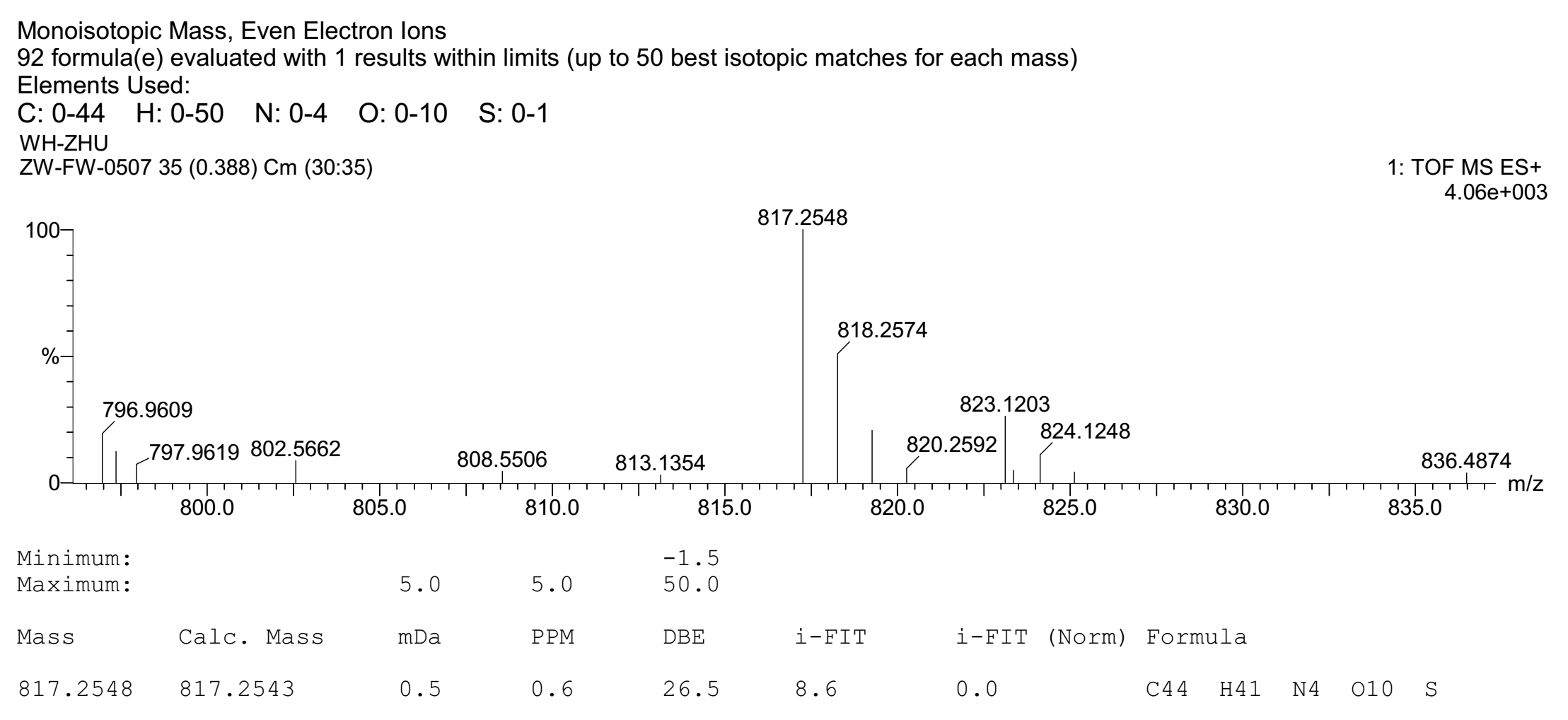


**Figure S21.** HRMS spectrum of QM-HBT-*β*galAc.


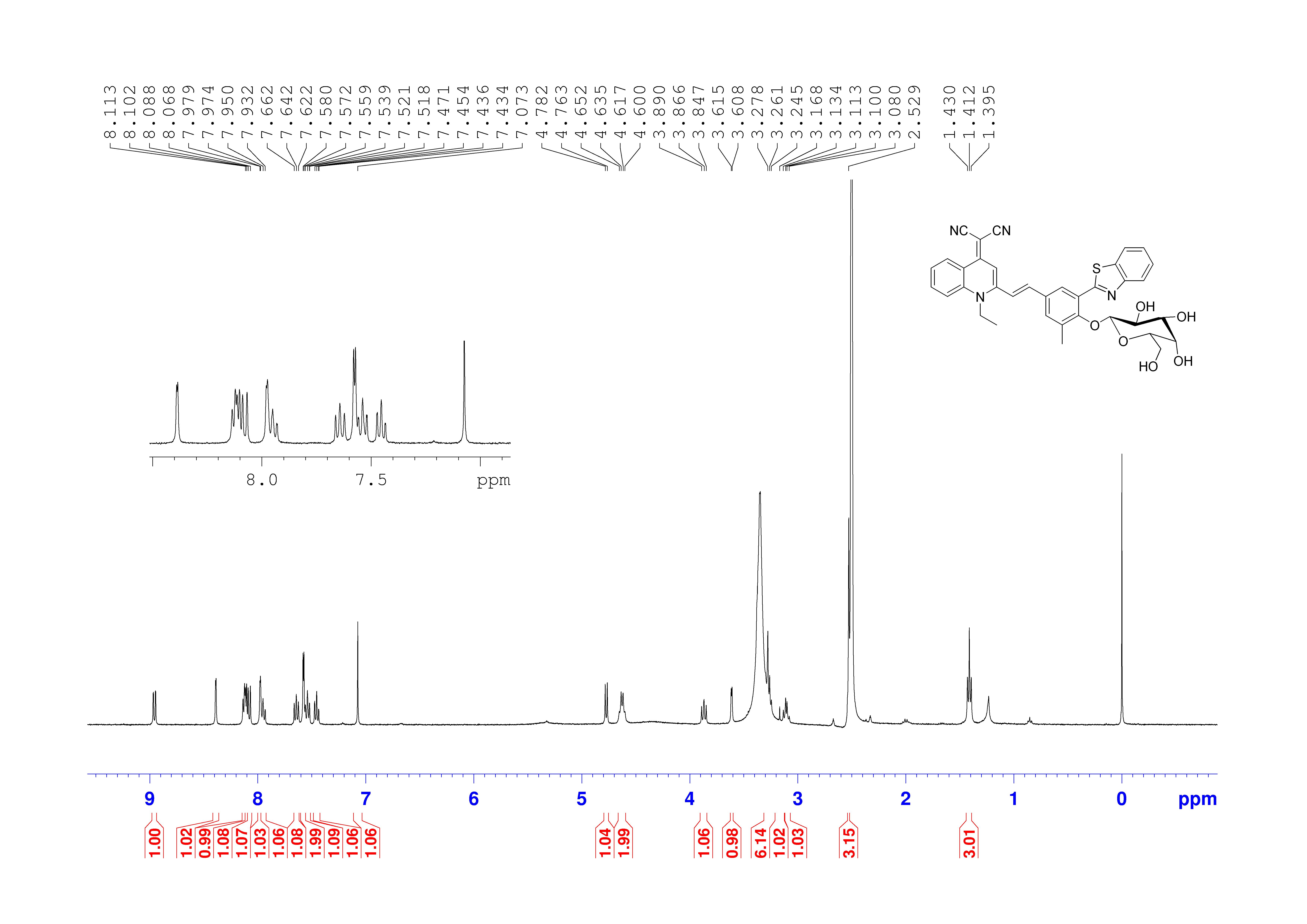


**Figure S22.** ^1^H NMR spectrum of QM-HBT-*β*gal DMSO-*d*_6_

_
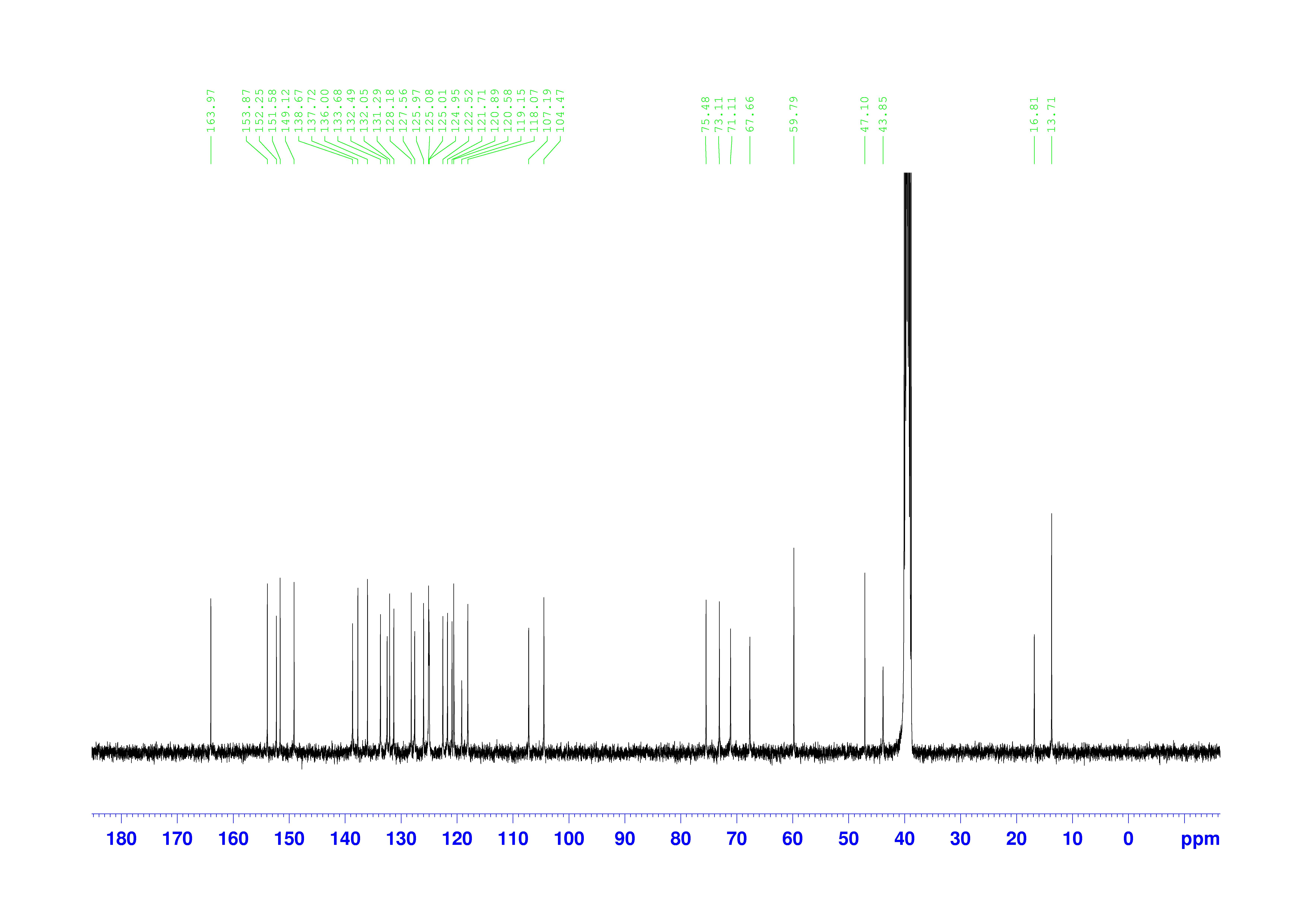
_

**Figure S23.** ^13^C NMR spectrum of QM-HBT-*β*gal in DMSO-*d*_6_


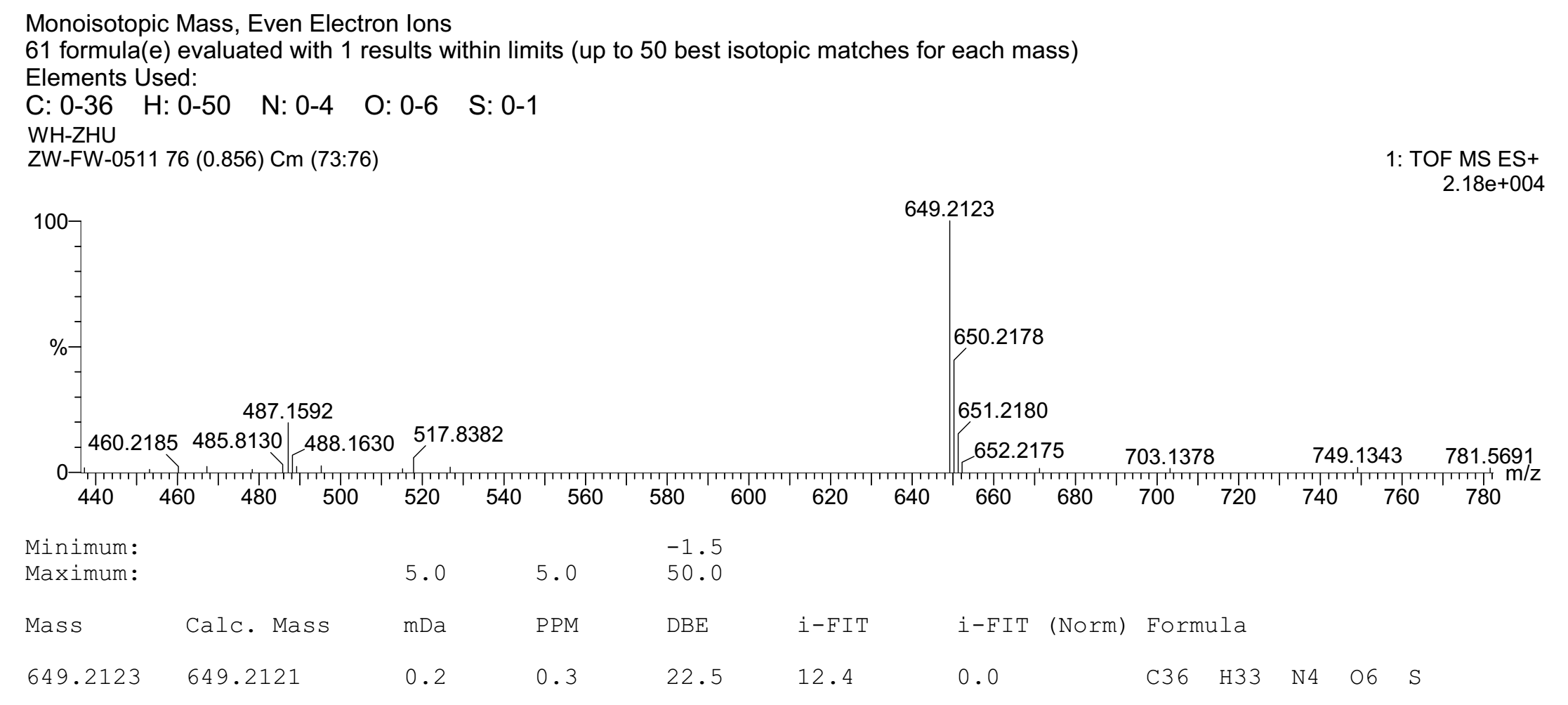


**Figure S24.** HRMS spectrum of QM-HBT-*β*gal.
